# Ketone body β-hydroxybutyrate ameliorates colitis by promoting M2 macrophage polarization through the STAT6-dependent signaling pathway

**DOI:** 10.1186/s12916-022-02352-x

**Published:** 2022-04-15

**Authors:** Chongyang Huang, Jun Wang, Hongbin Liu, Ruo Huang, Xinwen Yan, Mengyao Song, Gao Tan, Fachao Zhi

**Affiliations:** Guangdong Provincial Key Laboratory of Gastroenterology, Institute of Gastroenterology of Guangdong Province, Department of Gastroenterology, Nanfang Hospital, Southern Medical University, Guangzhou, 510515 China

**Keywords:** β-Hydroxybutyrate, Inflammatory bowel disease, M2 macrophage, STAT6, Tissue repair

## Abstract

**Background:**

Ketone body β-hydroxybutyrate (BHB) has received more and more attentions, because it possesses a lot of beneficial, life-preserving effects in the fields of clinical science and medicine. However, the role of BHB in intestinal inflammation has not yet been investigated.

**Methods:**

Colonic mucosa of inflammatory bowel disease (IBD) patients and healthy controls were collected for evaluation of BHB level. Besides, the therapeutic effect of exogenous BHB in a murine model of acute dextran sulfate sodium (DSS)-induced colitis were assessed by body weight change, colon length, disease activity index, and histopathological sections. The regulatory effectors of BHB were analyzed by RT-qPCR, immunofluorescence, and microbe analysis in vivo. Moreover, the molecular mechanism of BHB was further verified in bone marrow-derived macrophages (BMDMs).

**Results:**

In this study, significantly reduced BHB levels were found in the colonic mucosa from IBD patients and correlated with IBD activity index. In addition, we demonstrated that the administration of exogenous BHB alleviated the severity of acute experimental colitis, which was characterized by less weight loss, disease activity index, colon shortening, and histology scores, as well as decreased crypt loss and epithelium damage. Furthermore, BHB resulted in significantly increased colonic expression of M2 macrophage-associated genes, including IL-4Ra, IL-10, arginase 1 (Arg-1), and chitinase-like protein 3, following DSS exposure, suggesting an increased M2 macrophage skewing in vivo. Moreover, an in vitro experiment revealed that the addition of BHB directly promoted STAT6 phosphorylation and M2 macrophage-specific gene expression in IL-4-stimulated macrophages. Besides, we found that BHB obviously increased M2 macrophage-induced mucosal repair through promoting intestinal epithelial proliferation. However, the enhancement effect of BHB on M2 macrophage-induced mucosal repair and anti-inflammation was completely inhibited by the STAT6 inhibitor AS1517499.

**Conclusions:**

In summary, we show that BHB promotes M2 macrophage polarization through the STAT6-dependent signaling pathway, which contributes to the resolution of intestinal inflammation and the repair of damaged intestinal tissues. Our finding suggests that exogenous BHB supplement may be a useful therapeutic approach for IBD treatment.

**Supplementary Information:**

The online version contains supplementary material available at 10.1186/s12916-022-02352-x.

## Background

Inflammatory bowel diseases (IBD), including ulcerative colitis (UC) and Crohn’s disease (CD), are emerging as chronic relapsing inflammatory conditions in gastrointestinal tract, which are characterized by impaired intestinal barrier integrity and disorder of immune response with excessive pro-inflammatory cells infiltration while less generation of anti-inflammatory cells [1–3]. Signaling crosstalk between intestinal epithelial cells and immune cells deeply implicated in intestinal micro-environment homeostasis of IBD, among which macrophages are one of the most abundant cells in the intestine and closely involved in the pathogenesis of IBD [4].

Macrophages, as a core element of the innate immune systems, eliminate invading pathogen and can alter the polarization into pro-inflammatory (M1) and anti-inflammatory (M2) phenotypes dependent on the micro-environment [5–7]. As reported, M1 macrophages are elicited by lipopolysaccharide (LPS) and interferon-γ (IFN-γ) to promote Th1 immunity and produce pro-inflammatory cytokines for host defense against infection [8–11]. M2 macrophages are induced by IL-4 and IL-13 to modulate tissue remodeling, wound healing, and immune modulation through the IL-4 receptor alpha chain (IL-4Ra) [12–14]. Abnormal macrophage polarization occurs during the development of IBD, which has been recently targeted as a potential therapeutic strategy for IBD [4, 15, 16]. Macrophage polarization is regulated by various transcriptional factors. STAT1 mediates M1 activation, whereas STAT6 is responsible for M2 polarization [17]. However, how M2 macrophage polarization is fine-tuned by STAT6 is still incompletely clear.

The endogenous ketone metabolites refer to three kinds of small, water-soluble and lipid-derived molecules, consisting of acetoacetate, beta-hydroxybutyrate (BHB), and acetone [18–20]. Ketogenic enzymes, including acetyl-CoA-acetyltransferase-1 (ACAT1), 3-hydroxy-3-methylglutaryl-CoA-synthase-2 (HMGCS2), HMG-CoA lyase (HMGCL), and 3-hydroxybutyrate dehydrogenase-1 (BDH1), were highly expressed in the intestinal epithelium of healthy individuals [21]. Hence the ketone metabolic molecules are also largely produced in the gut of mammals during fatty acids oxidation metabolic program in time of nutrient deprivation, prolonged fasting, and exercise [18]. Accumulating evidence suggested that the most prominent ketone metabolite, β-hydroxybutyrate (BHB), possesses a lot of beneficial effects in the field of clinical science and medicine [22–24]. Previous studies reported that BHB presents the anti-aging effects during caloric restriction or fasting, which was generally considered to be beneficial to stem cell maintenance and tissue regeneration [18, 25, 26]. Recent studies also indicated that BHB can serve as important and instructive immune cell effectors through inhibiting Nlrp3 inflammasome activation and regulating intestinal pro-inflammatory Th17 cells [27–30]. However, whether BHB regulates M2 macrophage polarization in the intestine, inflammation, and IBD has remained elusive.

In this study, we explored the effects of BHB on human and experimentally induced IBD and elucidated the important roles of BHB in the pathogenesis of IBD. Here, we report that BHB levels are significantly decreased in the colonic mucosa from IBD patients and dextran sulfate sodium (DSS)-challenged mice. Importantly, the administration of exogenous BHB alleviates the severity of DSS-induced colitis. Moreover, BHB promotes M2 macrophage polarization through enhancing STAT6 phosphorylation induced by IL-4. Furthermore, we investigated the clinical significance of BHB in IBD patients and found that the levels of BHB in the colonic mucosa are negatively associated with the IBD activity index and the erythrocyte sedimentation rate (ESR) values in patients with IBD, which might offer an insight into the pathogenesis of IBD.

## Methods

### Antibodies and reagents

Anti-HMGCS2 (ab137043), anti-p-STAT6 (ab263947), anti-STAT6 (ab217998), and anti-p-JAK2 antibodies (ab92552) were obtained from Abcam. Anti-JAK2 antibody (3230T) was purchased from Cell Signaling Technology. Anti-ACAT1 (16215-1-AP) and anti-BDH1 (15417-1-AP) were purchased from Proteintech. Anti-ZO-1 (GB111981), anti-Occludin (GB111401), anti-F4/80 (GB113373), anti-Brdu (GB12051), and anti-PCNA (GB12010) were purchased from Servicebio.

Dextran sulfate sodium (DSS) was obtained from MP Biomedicals. Recombinant mouse GM-CSF (96-315-03-20) and recombinant mouse IL-4 (96-214-14-20) were obtained from Peprotech. BHB (S1031) and AS1517499 (S8685) were purchased from Selleck.cn. Cell Counting Kit-8 (CCK8) was obtained from Dojindo Laboratories, Japan. Clodronate liposomes (40337ES08) were obtained from Yeasen. Lipopolysaccharides (297-473-0), BHB sodium (13613-65-5), vancomycin (1404-93-9), neomycin-sulfate (1405-10-3), metronidazole (443-48-1), and ampicillin (69-52-3) were obtained from Sigma-Aldrich. BHB Colorimetric Assay Kit (700190) was obtained from Cayman Chemical. BCA Protein Assay Kit (P0012S) were obtained from Beyotime Biotechnology. Gentle Cell Disassociation Reagent (07174), DMEM/F12 with 15 mM HEPES (36254), and IntestiCult organoid growth medium (06005) were purchased from StemCell Technologies. Matrigel (356231) was purchased from Corning.

### Human samples

Endoscopic colonic mucosal biopsy samples were collected from IBD patients and non-IBD healthy donors attending the Department of Gastroenterology, Nanfang Hospital. All diagnoses and clinical disease activity assessments were based on a standard combination of clinical, endoscopic, histological, and radiologic criteria. All intestinal pinch biopsies were collected from consenting individuals during routine endoscopy according to the protocols approved by the Ethics Committee of Nanfang Hospital of Southern Medical University. Demographic characteristics are shown in Additional file 1: Table S2.

### Beta-hydroxybutyrate (BHB) measurements

Each colonic mucosal sample from human and murine model was split in two for total protein measurement and BHB measurement respectively. Samples were freshly washed by PBS and collected by centrifugation (centrifuged at 300×*g* for 5 min). Total proteins were measured by BCA Protein Assay Kit. BHB level was determined by BHB Colorimetric Assay Kit. Level of mucosal BHB was normalized to total protein of each sample.

### Mouse models

All animal research protocols were approved by the Institutional Animal Care and Use Committee of Southern Medical University (K2019032), which are in full compliance with the fundamental principles of replacement, reduction, and refinement in animal research. Wild type (WT) 8- to 10-week-old male C57BL/6 J mice (weighing 20–23 g) were obtained from Cyagen Biosciences and maintained under specific pathogen-free (SPF) condition in accredited animal facilities at Southern Medical University. Briefly, all mice were housed in a room with controlled temperature (21–23 °C) and lighting (12/12-h light-dark cycle) and were free access to water and food. Before samples collection, mice were anesthetized with an intraperitoneal injection of ketamine-medetomidine and euthanized by cervical dislocation. All efforts were made to minimize the suffering and discomfort experienced by the animals.

Mice model of DSS-induced colitis and regeneration was established according to a published procedure [31]. Briefly, mice were administered with water containing 2.5% DSS for 5 consecutive days, and then followed by 6 days normal drinking water, which was defined as regeneration after colitis. During experiment, body weight and disease activity index of mice were monitored every day. The scores of disease activity index (DAI) were evaluated according to the standard criteria provided by a published procedure [31]. The animals were included in the study according to the body weight change and DAI during DSS treatment. The animals were excluded if prematurely died preventing harvest of biological samples. The mice were sacrificed and colon tissues were collected for various analyses. Besides, histological scores of mice were assessed by two pathologists in a double-blind manner.

For exogenous BHB intervention, BHB enemas were prepared by using BHB sodium dissolved in saline. Mice were randomly divided into four groups (*n* = 7 to 10 per group): saline group, BHB group, saline DSS group, and BHB DSS group. Then mice were rectally administered with a single dose of BHB sodium solution (at the concentration of 15 mg/25 g) or saline as a control per day.

For intestinal macrophage depletion, mice were randomly divided into two groups (*n* = 7 to 8 per group): the saline DSS + clodronate group and the BHB DSS + clodronate group. Then, mice were intraperitoneally injected with clodronate liposomes (4 ml/kg) 5 days prior to DSS treatment and on days 2, 4, and 6 after the start of DSS treatment.

To remove intestinal microbiota, mice were randomly divided into two groups (*n* = 5 per group): the saline DSS + ABX group and the BHB DSS + ABX group. Then mice were intragastrically administered with antibiotics, including 100 mg/kg vancomycin, 200 mg/kg neomycin sulfate, 200 mg/kg metronidazole, and 200 mg/kg ampicillin, once a day for 5 days prior to DSS treatment.

To inhibit STAT6 phosphorylation in vivo, mice were randomly divided into two groups (*n* = 6 per group): saline DSS + AS group and BHB DSS + AS group. Then, mice were intraperitoneally injected with 5 mg/kg AS1517499 on days 1, 3, 5, 7, and 9 after the start of DSS treatment.

### Histochemistry and immunostaining

Colonic samples from people and mice were fixed in 4% paraformaldehyde for 24 h, embedded in paraffin, and 5-μm-thick sections were used for hematoxylin-eosin (H&E) or immunostaining. For immunohistochemistry and immunofluorescence staining, deparaffinized sections were performed quenching of endogenous peroxidase activity, antigen-retrieval, and subsequent blocking procedures. Slices were incubated in the primary antibodies at 4 °C overnight, followed by incubation with biotinylated secondary antibody for 2 h at room temperature. Protein expression levels were analyzed and scored by professional pathologists with Image-Pro Plus software.

### Isolation and culture of bone marrow-derived macrophages

Bone marrow-derived macrophages (BMDMs) were differentiated from bone marrow cells (BM cells) harvested from thighbone and shinbone from 8- to 10-week-old male WT mice. BM cells were cultured at 37 °C in 5% CO_2_ in Dulbecco's Modified Eagle Medium supplemented with 20% FBS, 100 U/mL penicillin, 100 μg/mL streptomycin, and 30 ng/ml GM-CSF-conditioned medium for 7 days to differentiate to BMDMs. Media were supplemented every 2 days. Non-adherent cells were removed by washing with PBS. After 7 days, the resultant adherent cells were harvested that consisted of > 95% F4/80^+^ macrophages.

For inflammatory macrophage differentiation, BMDMs were stimulated with 100 ng/mL LPS for 24 h. To generate anti-inflammatory macrophages, BMDMs were stimulated with 100 ng/mL recombinant IL-4 for 48 h. In LPS plus BHB group, BMDMs were treated with 100 ng/mL LPS and 5 mM BHB (S1031, selleck.cn) together for 24 h. In IL-4 plus BHB group, BMDMs were treated with 100 ng/mL IL-4 and 5 mM BHB together for 48 h. In rescue experiments, BMDMs were stimulated with 100 ng/mL IL-4, and then treated with 5 mM BHB or 100 nM AS1517499 alone or together for 48 h.

### Real-time PCR

Real-time PCR was performed as previously described [32]. The mRNA levels of target genes were normalized to that of GAPDH. The primers used for the target genes are shown in Additional file 1: Table S3.

### Western blot

Western blot analysis was performed as previously described [33]. GAPDH was used as an endogenous control. Anti-p-JAK2, anti-JAK2, anti-p-STAT6, and anti-STAT6 were diluted 1:1000. Anti-GAPDH was diluted 1:2000. Secondary antibodies were diluted 1:4000. The ImageJ software was used to quantify and analyze the density of the protein bands.

### RNA sequencing

RNA sequencing was used to measure the mRNA expression levels of many cytokines in IL-4-stimulated BMDMs with or without BHB.

### Colony-formation assay

Cell proliferation was measured using a cell counting kit-8 (CCK-8) according to the manufacturer’s instruction. Briefly, 90 μl of IEC-6 cells were plated at a concentration of 2000 cells/well in 96-well plates. CCK-8 working buffer (10 μl) was added into the cultures and incubated at 37 °C for 1 h. Colorimetric measurements were performed at 450 nm on a microplate reader.

### Mouse colon organoid culture

Mouse colon organoid was isolated and cultured according to the manufacturer's instruction. Briefly, the colons were obtained and luminal contents were flushed away by using cold PBS. The colons were then transferred to a biosafety cabinet and cut into 2–5 mm pieces. Colon pieces were further digested using the Gentle Cell Disassociation Reagent (StemCell Technologies) on a rocking platform for 20 min. The supernatant was removed and colon pieces were resuspended in cold PBS with 0.1% BSA. The new supernatant was then passed through a 70 -μm strainer and centrifuged at 4 °C 290×*g* for 5 min to obtain crypts. Isolated crypts were then resuspended in DMEM/F12 with 15 mM HEPES (StemCell Technologies) and counted under a microscope. An equal volume of IntestiCult Organoid Growth Medium (StemCell Technologies) and Matrigel (Corning) were mixed and the suspension was transferred into a pre-warmed 24-well-plate to form domes in the middle of each well. Complete IntestiCult Organoid Growth Medium was added above the domes, and crypts were cultured at 37 °C and 5% CO_2_. In addition, BMDMs were co-cultured with colon organoid in a transwell system with a 0.4-μm porous membrane that allows extracellular cytokines derived from the BMDMs to contact the organoids directly. Suppression of STAT6 phosphorylation was performed by treatment with 100 nM AS1517499 for 48 h in IL-4-stimulated BMDMs before co-culture.

### Organoid measurement

After 9 days in culture, all organoid numbers and budding organoids were manually counted in each well under light microscope to evaluate growth efficiency. For size measurement, four non-overlapping pictures of organoids in each well were captured under microscope. The surface area of organoid horizontal cross sections was then analyzed by using image-pro-plus software.

### Statistical analysis

Unless otherwise indicated, statistical analyses were performed using the GraphPad Prism software. Except as otherwise indicated, all experimental data are presented as the mean ± SEM, and statistical significance was determined using a two-tailed Student’s *t* test. *P-*values < 0.05 were considered significant.

## Results

### BHB generation are decreased in the colonic mucosa from active IBD patients

**Fig. 1:**
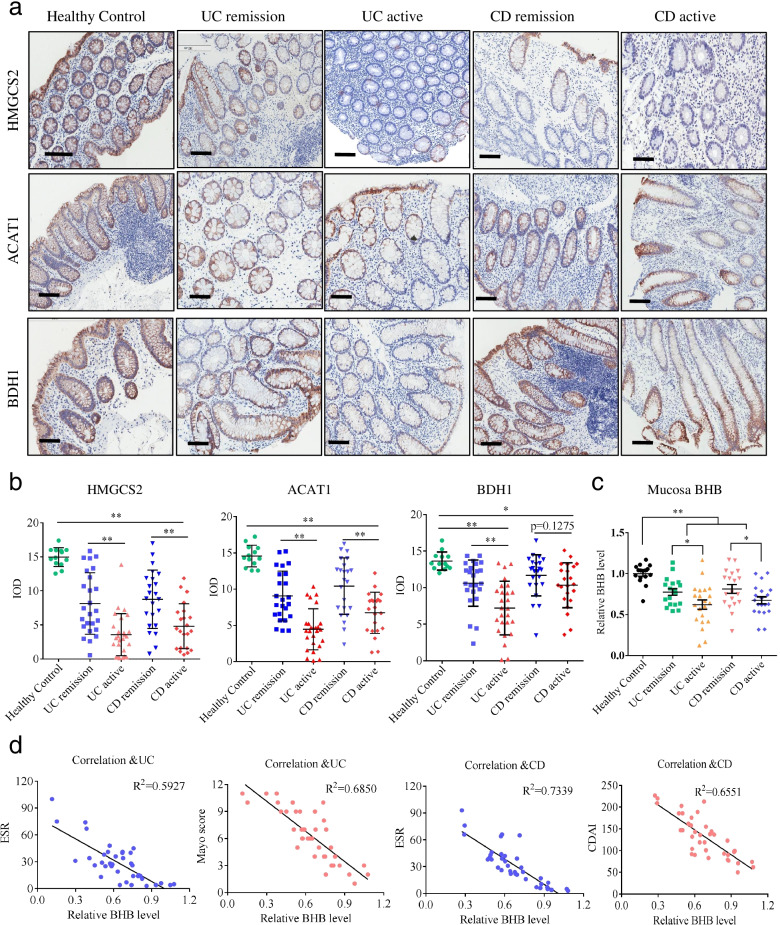
The clinical correlation of HMGCS2, ACAT1, BDH1, and BHB in IBD patients. UC, ulcerative colitis; CD, Crohn’s disease; ACAT1: acetyl-CoA acetyltransferase 1; HMGCS2, 3-hydroxymethylglutaryl-CoA synthase 2; BDH1, 3-hydroxybutyrate dehydrogenase I. IOD, integrated optical density (as measured by Image-pro-plus software). **a** Representative IHC images of the colonic mucosa from healthy controls and IBD patients in remission and active (scale bars: 150 μm). **b** IHC analyses of HMGCS2, ACAT1, and BDH1 protein in the colonic mucosa from healthy controls (*n* = 13), UC patients in remission (*n* = 25) and active (*n* = 26), and CD patients in remission (*n* = 23) and active (*n* = 22). **c** Enzymatic colorimetric assay of BHB levels in the colonic mucosa from healthy controls (*n* = 16), UC patients in remission (*n* = 18) and active (*n* = 21), and CD patients in remission (*n* = 19) and active (*n* = 20). **d** Correlation between BHB levels and the Mayo scores in UC and the CDAI scores in CD and the ESR values in IBD were calculated by Spearman correlation analyses. **b**, **c** Data represent means ± SEM; *P< 0.05, **P< 0.01 by unpaired Student’s *t* test

Given the evident significance of ketogenic enzyme in ketogenesis, we first analyzed the expression levels of genes encoding ketogenic enzymes in public datasets of IBD samples and found that the mRNA levels of *ACAT1*, *HMGCS2*, and *BDH1* were significantly decreased in the colonic mucosa from active IBD patients compared to healthy controls, while there was not significant difference in the mRNA level of *HMGCL* [34]. Consistently, we found that the protein levels of *ACAT1*, *HMGCS2*, and *BDH1* were significantly reduced in the colonic mucosa from active IBD patients compared to healthy controls (Fig. 1a, b). In addition, DSS mice had decreased expression of *ACAT1*, *HMGCS2*, and *BDH1* in the colonic tissues (Additional file 1: Fig. S1a-c). These findings suggest that IBD patients may have reduced endogenous ketone metabolites in the colon.

Since BHB is the most prominent ketone metabolite, we subsequently detected the level of BHB by β-Hydroxybutyrate colorimetric assay kit and found that the level of BHB was significantly decreased in the colonic mucosa from active IBD patients compared to healthy controls (Fig. 1c). Furthermore, we explored the clinical impact of BHB in IBD patients and found that colonic BHB levels were negatively correlated with the IBD activity index (Mayo scores in the UC and CDAI in the CD) and ESR values (Fig. 1d). Taken together, these results suggest that decreased BHB production in the colon might be involved in the pathogenesis of IBD.

### Exogenous BHB supplement attenuates DSS-induced colitis

To assess whether BHB participates into experimental colitis, we first conducted a study on the colonic level of BHB in mice challenged with 2.5% DSS in drinking water. BHB levels were markedly decreased in the colonic tissues after 6-day DSS treatment (Fig. S1d). This result suggests that BHB may be involved in the development of colitis.

**Fig. 2:**
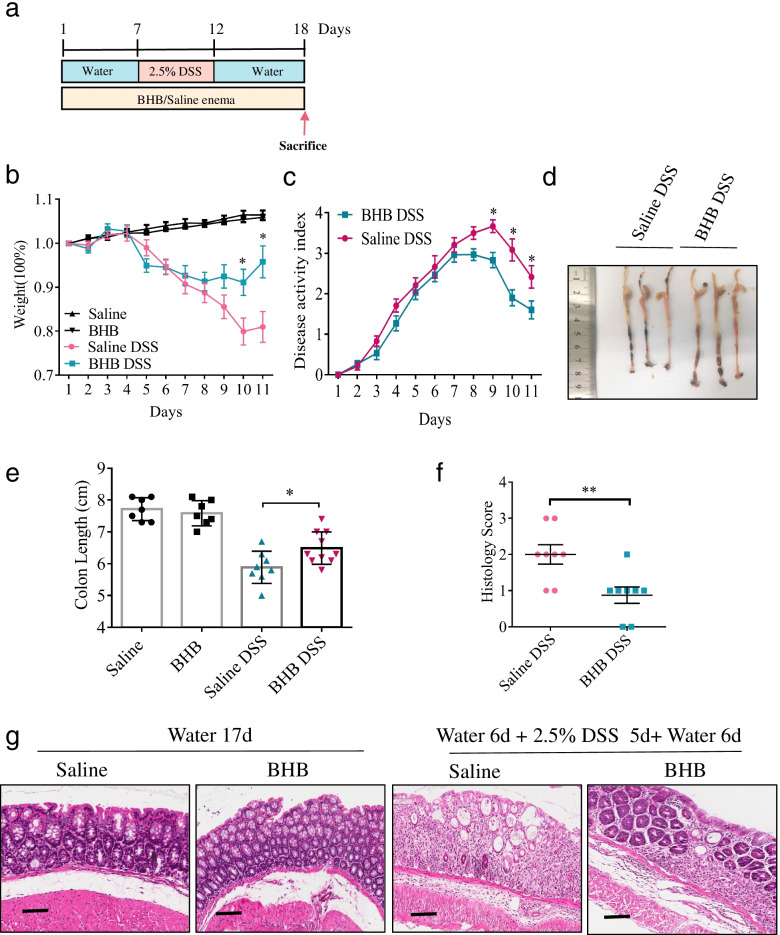
Exogenous BHB supplement alleviates DSS-induced colitis. **a**–**g** Littermate WT mice (*n* = 7 to 10 per group) were fed with drinking water for 6 days, then 2.5% DSS in drinking water for 5 days, and followed by drinking water for 6 days, or with drinking water alone for 17 days. These mice were rectally injected with BHB enema or saline as a control. **b**, **c** Body weight changes and disease activity index were monitored daily after DSS administration. **d** Representative images of the colons from BHB-treated mice and controls. **e** Mice were killed on day 18, and colon lengths were measured. **f**, **g** Colon sections were examined histologically. **f** Histology scores for colonic inflammation were measured. **g** Representative images of the H&E-stained colon sections of different treatment groups (scale bars: 150 μm). **b**, **c**, **e**, **f** Data represent means ± SEM; *P< 0.05, **P< 0.01 by unpaired Student’s *t* test. Data shown are representative of three independent experiments

To further confirm the role of BHB in DSS-induced colitis, we next rectally injected exogenous BHB or saline as a control to DSS-exposed mice, according to the methods shown in Fig. 2a. BHB-treated mice showed significantly reduced weight loss, disease activity index, and colon shortening, compared to control mice (Fig. 2b–e). Moreover, histological examination displayed less crypt loss, epithelium damage, and decreased inflammation in the colons of BHB-treated mice than in those of control mice (Figs. 1g and 2f). However, without DSS exposure, BHB administration had no effect on body weight, colonic length, and histology (Fig. 2b, e, g). These results indicate that BHB protects mice from DSS-induced colitis may through inhibiting colonic inflammation and promoting post-injury repair of colonic tissues.

### BHB exerts protective effect against colitis dependently via colonic macrophages

Since intestinal epithelial barrier, gut microbiota, and macrophages are critical for the pathological processes associated IBD, we investigated whether the beneficial effect of BHB on colitis is dependent on these factors. First, we examined whether BHB affects intestinal epithelial integrity by measuring the levels of immunofluorescence staining of tight junction proteins ZO-1 and occluding. No difference was noted in ZO-1 and occluding immunostaining between BHB-treated and control groups (Additional file 1: Fig. S2a-d).

The 16S diversity analysis showed that there was no significant difference in alpha diversity between the two groups of mice (Additional file 1: Fig. S3a-b). However, the relative abundance of specific strains, such as *Firmicutes* at the phylum level and *Lachnospiraceae* at the family level, was significantly increased in the BHB group when compared to that in the saline group (Additional file 1: Fig. S3c-e); unweighted PCoA analysis revealed that clusters of gut microbiota between BHB and saline mice were entirely separated, suggesting that the gut flora exhibited a very different composition in two groups (Additional file 1: Fig. S3d).

Therefore, we explored whether BHB intervention attenuates DSS-induced colitis is dependent on gut microbiota. To this end, mice were treated with antibiotics (ABX) to ablate the impact of intestinal microbiota followed by rectally injecting exogenous BHB or saline as a control to DSS-exposed mice (Additional file 1: Fig. S4a). After ABX treatment, the mice received BHB enema still developed decreased colitis as reflected by less body weight loss, disease activity index, and colon shortening, as well as significantly reduced histology scores, compared to control mice (Additional file 1: Fig. S4b-g). These results indicate that BHB ameliorates DSS-induced colitis independently of intestinal microbiota.

**Fig. 3:**
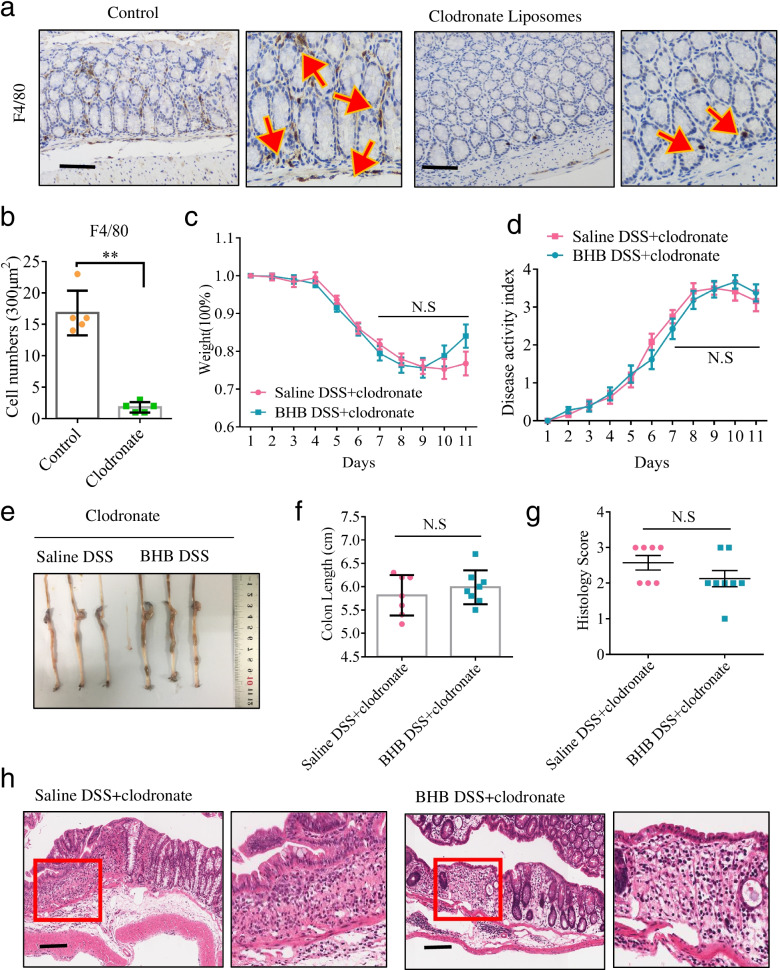
The protective effect of BHB against colitis is depending on colonic macrophages. **a**, **b** Littermate WT mice (*n* = 5 per group) were intraperitoneally injected with clodronate liposomes (4 ml/kg) or PBS as a control once a day for 5 days. Their colonic tissues were collected for IHC analyses to determine the efficiency of macrophage depletion. **a** Representative IHC images of F4/80 immunostaining in the colon tissues (scale bars: 150 μm). **b** The number of colonic F4/80^+^ immune cells per 300 μm^2^. F4/80^+^ cells were counted under a microscope. **c**–**h** Littermate WT mice (*n* = 7 to 8 per group) received BHB or saline enema were given DSS in drinking water to induce experimental colitis as described in Fig. 1a. For macrophage depletion, clodronate liposomes (4 ml/kg) were intraperitoneally injected once a day for 5 days prior to DSS treatment and on days 2, 4, and 6 after the start of DSS treatment. **c**, **d** Body weight changes and disease activity index were monitored daily after DSS treatment. **e** Representative images of the colons. **f** Mice were killed on day 18, and colon lengths were measured. **g**, **h** Colon sections were examined histologically. **g** Histology scores for colonic inflammation were measured. **h** Representative images of the H&E-stained colon sections of different treatment groups (scale bars: 150 μm). **b**–**d**, **f**, **g** Data represent means ± SEM; NS, not significant; **P< 0.01 by unpaired Student’s *t* test. Data shown are representative of three independent experiments

Subsequently, we asked whether intestinal macrophages are involved in the protective effect of BHB during colitis development. To test it, mice were intraperitoneally injected with clodronate liposomes to accomplish macrophage depletion (Fig. 3a, b). Notably, ablation of macrophage largely abrogated the protective effect of BHB, which failed to improve the clinical parameters of DSS-induced colitis such as body weight loss, disease activity index, and colon shortening (Fig. 3c–f). Histological examination further demonstrated that the beneficial effect of BHB was largely diminished when deletion of intestinal macrophage (Fig. 3g, h). These results show that the protective effect of BHB against DSS-induced colitis is depend on intestinal macrophages.

### The protective effect of BHB against colitis involves M2 macrophage polarization

**Fig. 4:**
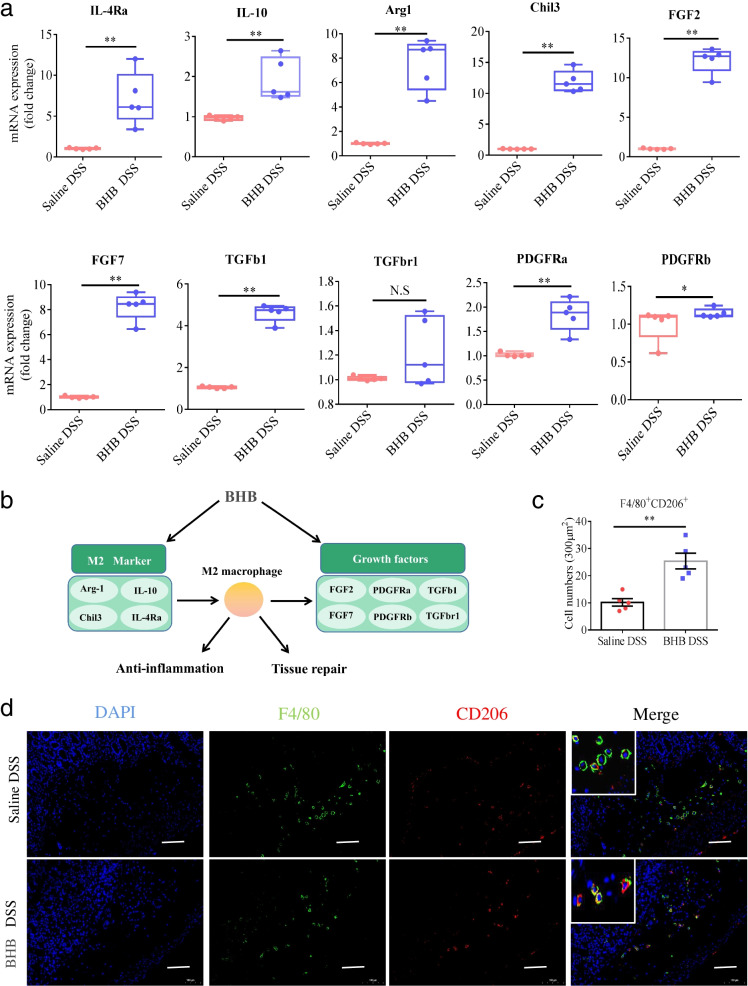
BHB increases colonic M2 macrophages in DSS-induced colitis. Littermate WT mice received BHB or saline enema were given DSS in drinking water to induce experimental colitis as described in Fig. 1a. On day 18, these mice were killed and their colon tissues were collected for the following analyses. **a** Real-time PCR analyses of indicated genes in whole-colonic homogenates. The mRNA levels of target genes were normalized to that of GAPDH. **b** Systematic diagram of anti-inflammatory M2 macrophage and related biological functions potentially correlated with the 10 upregulated genes. **c** The number of colonic F4/80^+^ CD206^+^ immune cells per 300 μm^2^. M2 macrophages were co-immunostained with F4/80 (green) and CD206 (red). F4/80^+^ CD206^+^ cells were counted under a microscope. **d** Representative immunofluorescence images of F4/80 and CD206 immunostaining in colon tissues (scale bars: 100 μm). White boxes represent the magnified view. **a**, **c** Data represent means ± SEM (*n* = 5 per group); NS, not significant; *P< 0.05, **P< 0.01 by unpaired Student’s *t* test. Data shown are representative of three independent experiments

A previous study reported that BHB reduces neuroinflammation through shaping microglia, a group of ramified brain-resident phagocytes, toward anti-inflammatory M2 polarization [35]. Thus, we proposed that BHB administration attenuates DSS-induced colitis may through regulating M2 macrophage polarization in the intestine. As expected, BHB administration resulted in significantly increased mRNA expression of M2 macrophage-associated genes including IL-4Ra, IL-10, arginase 1 (Arg-1), and chitinase-like protein 3 (Chil3), and their downstream effectors, such as FGF2, FGF7, TGFb, TGFbr1, PDGFra, and PDGFrb in the colons of DSS-exposed mice (Fig. 4a, b). Moreover, BHB significantly increased in the number of F4/80^+^CD206^+^ M2 macrophages in the colons of DSS-exposed mice as determined by immunofluorescence staining (Fig. 4c, d). Taken together, these results indicate that BHB protects mice from DSS-induced colitis through enhancing M2 macrophage polarization.

### BHB promotes IL-4-induced M2 macrophage polarization through the JAK2-STAT6 signaling pathway

**Fig. 5:**
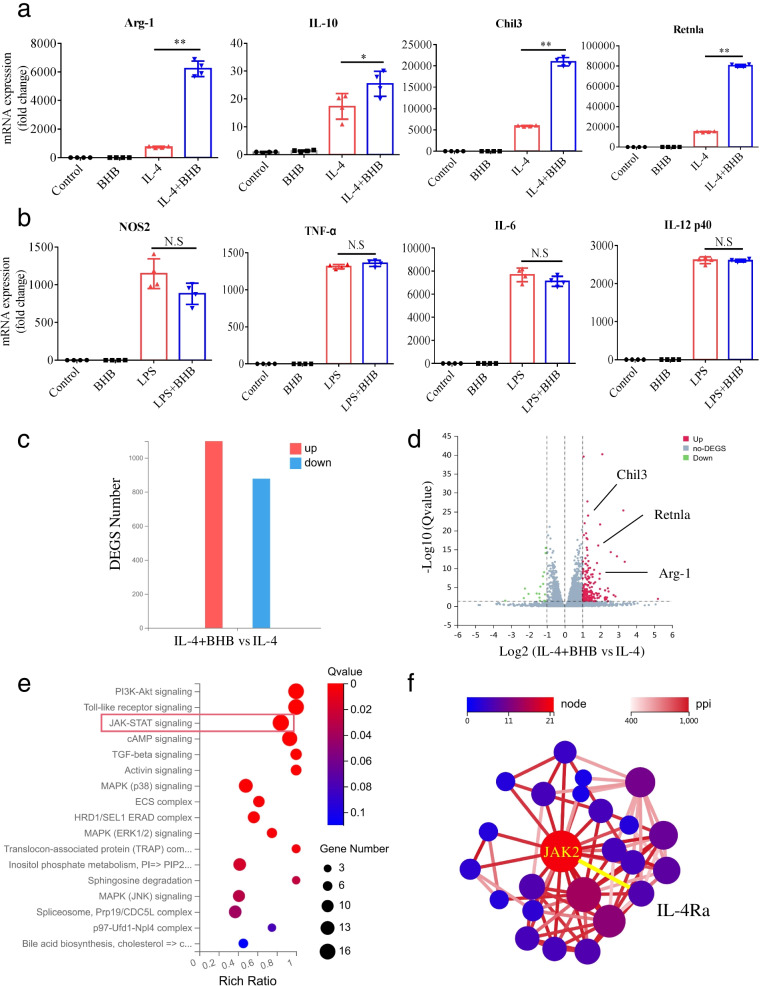
BHB promotes IL-4-induced M2 macrophage polarization in vitro. **a** BMDMs isolated from mice were stimulated with BHB or IL-4 alone or together. Forty-eight hours later, the relative mRNA expression levels of the indicated genes were determined by real-time PCR and normalized to GAPDH. **b** BMDMs isolated from mice were stimulated with BHB or LPS alone or together. Twenty-four hours later, the relative mRNA expression levels of the indicated genes were determined by real-time PCR and normalized to GAPDH. **c**–**f** BMDMs isolated from mice were stimulated with IL-4 or IL-4 plus BHB. Forty-eight hours later, differentially expressed genes (DEGS) were analyzed by RNA-sequencing. **c** The number of DEGS in the IL-4 plus BHB group vs the IL-4 group. Red represented upregulated DEGS and blue downregulated DEGS. **d** Scatter plot showing DEGS in the IL-4 plus BHB group vs the IL-4 group. Genes were plotted based on their expression levels (log10 intensity). Red and green dots represented up- and downregulated genes, respectively. M2 macrophage-specific genes were indicated. **e** KEGG pathway enrichment analyses of the upregulated DEGS. Dot size represents the number of DEGS, and the dot color represents the corresponding *p* value. **f** Protein–protein interaction (PPI) analyses of the upregulated DEGS. **a**, **b** Data represent means ± SEM from four independent experiments; NS, not significant; *P< 0.05, **P< 0.01 by unpaired Student’s *t* test

To confirm the function of BHB on the induction of M2 macrophage polarization, BHB was added to bone marrow-derived macrophages (BMDMs) isolated from mice in vitro. In agreement with the in vivo data, the addition of BHB significantly increased the mRNA expression of M2-associated genes, including Arg-1, IL-10, Chil3, and Retnla, in IL-4-stimulated BMDMs, but not in BMDMs (Fig. 5a). However, BHB addition did not affect the mRNA expression of M1-associated genes, such as NOS2, TNF-α, IL-6, and IL12p40, in BMDMs, nor in LPS-stimulated BMDMs (Fig. 5b). These results show that BHB promotes IL-4-induced M2 macrophage polarization.

To further dissect the underlying mechanism, BMDMs isolated from mice were stimulated with IL-4 alone or with IL-4 plus BHB, and the mRNA expression profiles were analyzed by RNA sequencing. We observed that a large number of genes, including M2 macrophage-specific genes Arg-1, Chil3, and Retnla, were upregulated in the IL-4 plus BHB group compared to the IL-4 group (Fig. 5c, d). With Kyoto Encyclopedia of Genes and Genomes (KEGG) pathway enrichment analysis, it was found that these upregulated genes were significantly enriched in regulation of the JAK-STAT signaling pathway (Fig. 5e). Moreover, protein–protein interaction network analysis of these upregulated genes identified Janus kinase 2 (JAK2) as a downstream signaling molecule of IL-4Ra (Fig. 5f). Binding of IL-4 to IL-4Rα results in tyrosine phosphorylation and activation of STAT6, which is a key signaling node for M2 macrophage polarization [36, 37]. Our findings combined with previous findings suggest that BHB promotes M2 macrophage polarization may through the JAK2-STAT6 signaling pathway.

**Fig. 6:**
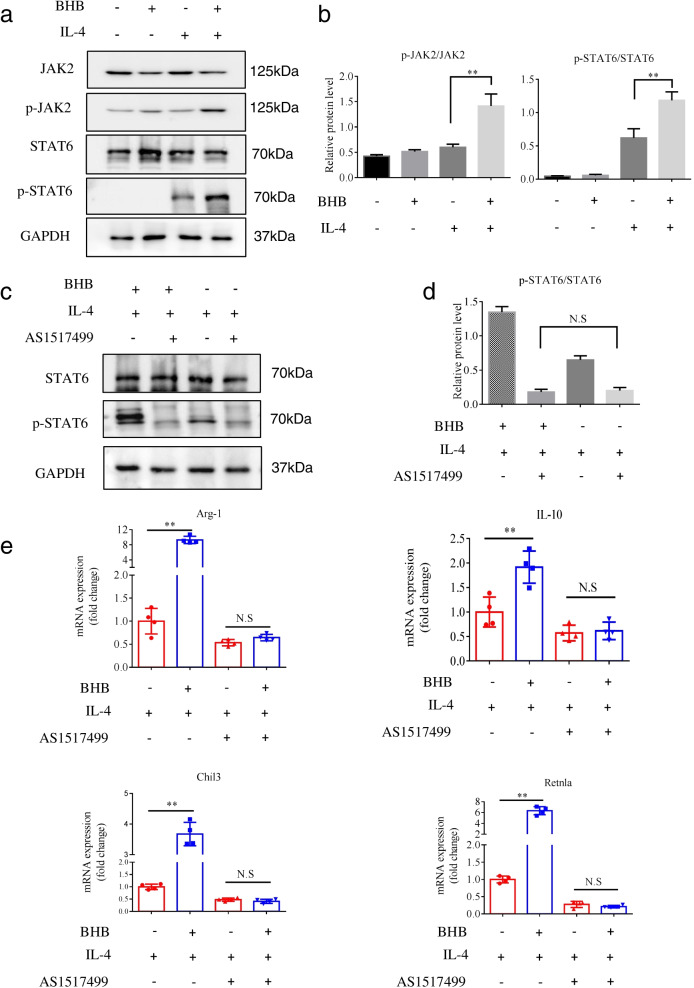
BHB promotes IL-4-induced M2 macrophage polarization through the STAT6-dependent signaling pathway. **a**, **b** BMDMs isolated from mice were stimulated with BHB or IL-4 alone or together. Forty-eight hours later, the protein expression of the indicated genes was determined by western blot. **a** Representative immunoblot images of p-JAK2, JAK2, p-STAT6, and STAT6 in different treatment groups. **b** Relative protein levels of p-JAK2/JAK2 and p-STAT6/STAT6 in different treatment groups. The density of protein bands was quantified by ImageJ software. **c**–**e** BMDMs isolated from mice were stimulated with IL-4, and then treated with BHB or AS1517499 alone or together. Forty-eight hours later, these treated cells were collected for the following analyses. **c** Representative immunoblot images of p-STAT6 and STAT6 in different treatment groups. **d** Relative protein levels of p-STAT6/STAT6 were determined by western blot and quantified by Image-pro-plus software. **e** Relative mRNA levels of M2-associated genes Arg-1, IL-10, Chil3, and Retnla were determined by real-time PCR and normalized to GAPDH. Data represent means ± SEM from three (**b** and **d**) and four (**e**) independent experiments; NS, not significant;**P< 0.01 by unpaired Student’s *t* test

We next performed western blot analysis to examine whether BHB influences the activation of the JAK2-STAT6 signaling pathway in BMDMs. STAT6 phosphorylation was undetected in unstimulated BMDMs or in stimulated cells with BHB alone. As expected, obvious STAT6 phosphorylation was observed in IL-4-stimulatedBMDMs (Fig. 6a). The addition of BHB increased the intensity of phosphorylated STAT6, but not total STAT6, in IL-4-stimulated BMDMs (Fig. 6a). This result was further confirmed by a significant increase in pSTAT6 when normalized to total STAT6 (Fig. 6b). Consistently, IL-4 and BHB together resulted in significantly increased JAK2 phosphorylation, but both alone cannot in BMDMs (Fig. 6a, b). Collectively, these results indicate that BHB enhances IL-4-induced activation of the JAK2-STAT6 signaling pathway in BMDMs.

We subsequently examined whether the upregulation effect of BHB on IL-4-induced M2 macrophage polarization is dependent on the JAK2-STAT6 signaling pathway. To this end, AS1517499 (AS), a potent STAT6 phosphorylation inhibitor, was administrated in IL-4-stimulated BMDMs with or without BHB, and the mRNA expression of M2-associated genes was measured by real-time PCR. We found that AS treatment effectively suppressed STAT6 phosphorylation in IL-4-stimulated BMDMs (Fig. 6c, d). Moreover, the upregulation effect of BHB on IL-4-induced STAT6 phosphorylation was obviously inhibited by AS (Fig. 6c, d). Consistently, BHB significantly increased the mRNA levels of Arg-1, IL-10, Chil3, and Retnla in IL-4-stimulated BMDMs, but this upregulation role was completely inhibited by AS (Fig. 6e). Taken together, these results show that BHB could directly promote IL-4-induced M2 macrophage polarization through enhancing the JAK2-STAT6 signaling pathway.

### The protective effect of BHB against colitis is dependent on STAT6 activation

Because STAT6 activation is a critical signaling node for BHB’s regulating M2 macrophage polarization, we speculated that it is also required for BHB therapeutic role during colitis development. To test it, BHB enema or saline as a control was rectally injected to DSS-exposed mice, then these mice were received intraperitoneal injection of AS1517499 (Additional file 1: Fig. S5a). The mice received BHB treatment displayed increased p-STAT6 phosphorylation in F4/80^+^ macrophages compared to control mice (Additional file 1: Fig. S6a and c), which was effectively inhibited by AS administration in vivo (Additional file 1: Fig. S6b and d). Consistently, AS administration largely eliminated the protective effect of BHB against DSS-induced colitis as reflected by no significant difference in weight loss, disease activity index, colon shortening, and histology scores between the BHB and saline groups (Additional file 1: Fig. S5b-g). These results indicate that the protective effect of BHB against DSS-induced colitis is dependent on the STAT6 signaling pathway.

### BHB-treated M2 macrophages promote intestinal epithelial regeneration

**Fig. 7:**
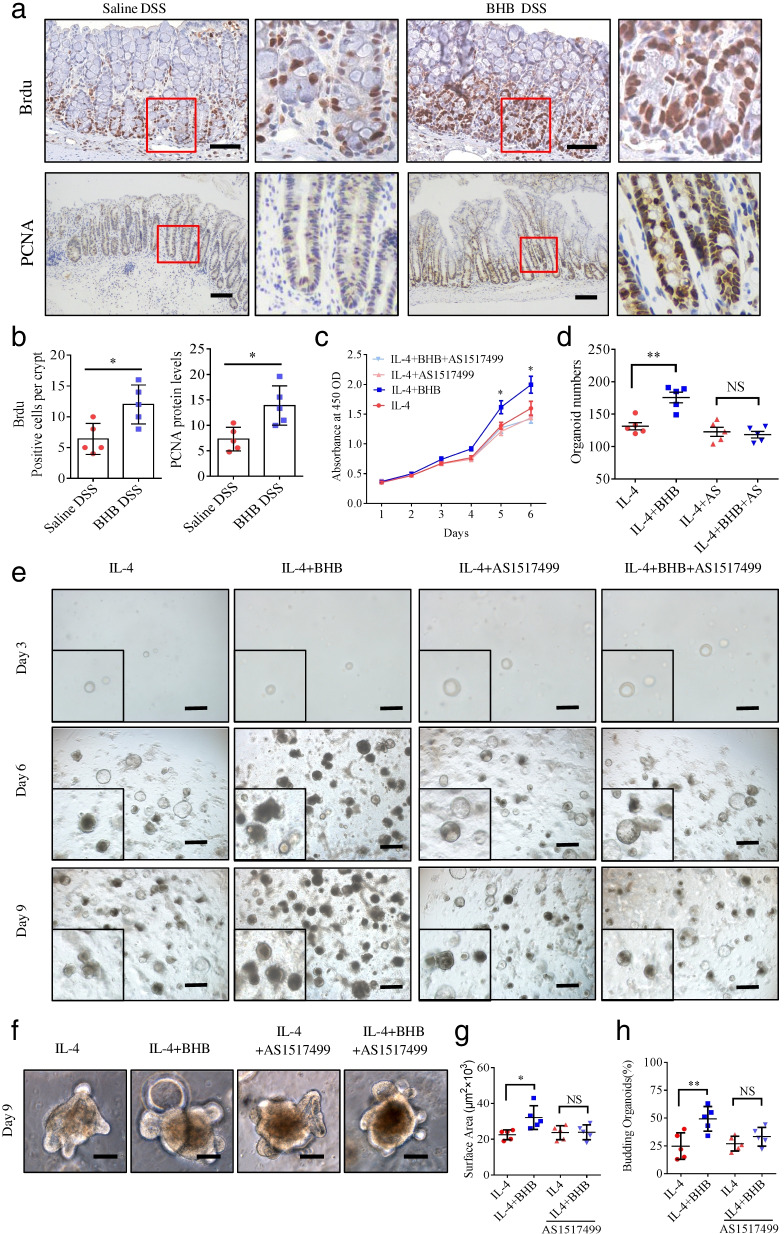
BHB-treated M2 macrophages promote intestinal epithelial cell proliferation. **a**, **b** Littermate WT mice received BHB or saline enema were given DSS in drinking water to induce experimental colitis as described in Fig. 2a. On day 18, these mice were killed and their colon tissues were collected for IHC analyses. **a** Representative IHC images of Brdu and PCNA immunostaining in the colon tissues (scale bars: 150 μm). **b** Quantitative analyses of Brdu and PCNA staining by Image-Pro-Plus software. Data represent means ± SEM from 6 mice in each group. **c**–**e** BMDMs isolated from mice were stimulated with IL-4, and then treated with BHB or AS1517499 alone or together. Forty-eight hours later, the cell culture supernatant purified by ultrafiltration was used to stimulate IEC-6 cells (**c**), and the cells were co-cultured with colon organoids in a transwell system (**d**, **e**). **c** IEC-6 cell viability was measured by CCK-8 assay. Data represent means ± SD from quintuplicate wells in each group. **d** Colon organoids were harvested and counted after 9-day supernatant stimulation. Data represent means ± SEM from three independent experiments. **e** Representative images of colon organoids after 3-, 6-, and 9-day supernatant stimulation (scale bars: 300 μm). **f** Representative images of colon organoids after 9-day supernatant stimulation (scale bars: 50 μm).**g** Quantitative analyses of average size of (day 9) colonic organoids. **h** Quantitative analyses of new crypt formation (budding) of (day 9) organoids. NS, not significant; *P < 0.05, **P < 0.01 by unpaired Student’s *t* test. Data shown are representative of three (**a**–**c**) independent experiments

Previous studies have shown that M2 macrophage contributes to tissue repair and regeneration [38, 39]. In this study, we found that BHB treatment promotes intestinal epithelial proliferation during colitis development (Fig. 7a, b). Therefore, we evaluated whether BHB regulates M2 macrophage function in intestinal epithelial proliferation. To test it, BMDMs isolated from mice were stimulated with IL-4 alone or with IL-4 and BHB together to induce M2 macrophage polarization. Then, the cell culture supernatant purified by ultrafiltration was used to stimulate the normal intestinal epithelial cell line IEC-6 and cell proliferation was measured by CCK-8 assays. We found that the levels of cell proliferation were significantly higher in the IL-4 plus BHB group than in the IL-4 group (Fig. 7c). However, this effect was completely inhibited by AS1517499 (Fig. 7c).

To mimic the in vivo environment and investigate the effect of BHB-treated M2 macrophages on intestinal epithelium proliferation, mouse colonic crypts were isolated and cultured in vitro. Then, we co-cultured these colonic organoids with IL-4-stimulated BMDMs with or without BHB in a transwell system, which allows extracellular cytokines derived from the BMDMs to contact the organoids directly. We found that organoids grew without gross abnormalities in the presence of IL-4. Moreover, the number of colonic organoids were significantly higher in the IL-4 plus BHB group than in the IL-4 group (Fig. 7d, e). Moreover, in the IL-4 plus BHB group, organoids were larger in size and show higher percentage of buddings than that in the IL-4 group (Fig. 7f–h). However, these effects were obviously inhibited by AS1517499 (Fig. 7d–h). Taken together, these results indicate that BHB-treated M2 macrophages promote intestinal epithelial cells proliferation and organoids growth through the STAT6-dependent signaling pathway.

## Discussion

Ketone body BHB is generally considered as source of energy for tissues during periods of low-energy states or dietary deprived of carbohydrates [40]. BHB has received more and more attentions, because it possesses a lot of beneficial, life-preserving effects in the fields of clinical science and medicine. However, the role of BHB in intestinal inflammation has not yet been investigated. We herein demonstrate that BHB alleviates intestinal inflammation in DSS-induced colitis, which could be due to its ability to promote M2 macrophage polarization. Furthermore, we demonstrate that locally BHB levels are negatively correlated with the severity of DSS-induced colitis and human IBD.

M2 macrophage polarization has been proposed to play important roles in IBD [15, 41]. Activated macrophages with M2 phenotype results in increased resistant to DSS-induced colitis [42], while adoptive transfer of in vitro-derived M2 macrophages reduces colonic inflammation in mice [43]. In addition, patients with active CD have reduced numbers of M2-like macrophages, whereas patients with inactive CD have increased numbers of these cells [43]. Therefore, we hypothesized that BHB may regulate M2 macrophage skewing in DSS-exposed colons. Indeed, we found that BHB treatment lead to significantly increased expression of M2-associated genes in DSS-exposed colons, suggesting locally increased M2 macrophage responses. Moreover, we found that BHB was able to directly promote M2 macrophage polarization in vitro, as the addition of BHB markedly increased the expression of M2-associated genes in IL-4-stimulated BMDMs, as well as the phosphorylation of STAT6, which is crucial for M2 macrophage polarization [36]. A previous study showed that macrophage polarization through IL-4 can occur in joint inflammation [44]. It is also possible that BHB could switch M1 macrophages into M2 macrophages in the presence of IL-4 in colonic inflammation, whereas whether this is indeed the case needs to be further investigated.

Although we found that BHB increased STAT6 phosphorylation in IL-4-stimulated BMDMs, BHB alone did not activate STAT6 in BMDMs, suggesting that BHB synergistically enhances IL-4-induced STAT6 phosphorylation and subsequent M2 polarization. Furthermore, we found that BHB increased JAK2 phosphorylation in the presence of IL-4. Binding of IL-4 to IL-4Rα results in signal transduction through JAK2 and phosphorylation of STAT6 [45]. These findings indicate that BHB promotes macrophage M2 polarization through facilitating the JAK2/STAT6 signaling pathway. Protein lysine acetylation is a dynamic process, in which an acetyl group is transferred to a specific lysine by an acetyltransferase like CBP, p300, and PCAF [36]. A recent study has demonstrated that STAT6 acetylated by CBP negatively modulates macrophage M2 polarization [36]. It is possible that BHB promotes macrophage M2 polarization through downregulating CBP-mediated STAT6 acetylation. However, the exact molecular mechanism remains to be further studied.

In this study, we show that BHB facilitates mucosal repair through promoting intestinal epithelial proliferation in murine IBD. Using the DSS-induced colitis model that mimics the clinical pathology of IBD, we found that BHB-treated mice had less crypt loss and epithelium damage in the DSS-exposed colons. Furthermore, we found that BHB increased the expression of bromodeoxyuridine (Brdu) and proliferating cell nuclear antigen (PCNA), two cell proliferation markers, in the DSS-exposed colons. Because M2 macrophages accelerate tissue repair [39], we speculated that BHB promotes intestinal epithelial proliferation via regulating M2 macrophages. Indeed, we found that BHB-treated M2 macrophages effectively promoted the proliferation of IEC-6 and colonic organoids, but the improvement was significantly inhibited by the STAT6 inhibitor AS1517499.

M2 macrophages are frequently termed “wound healing” macrophages as they produce factors that are important for tissue repair [41]. Clearly, M2 macrophages produce TGFb, Arg-1, and Wnt ligands, which are known to be involved in cell proliferation and tissue repair [41, 46]. TGFb enhances fibroblast proliferation and collagen production observed in macrophage/fibroblast co-cultures [46]. Arginine metabolism by Arg-1 produces polyamines, which are important for cell proliferation [47]. Macrophage Wnt7b contributes to kidney repair through stimulating epithelial responses [48]. Here, we found that BHB significantly increased the expression of Arg-1, TGFb2, TGFb3, Wnt5a, and Wnt10b in IL-4-stimulated BMDMs, among which Arg-1 is the most upregulated gene (Additional file 1: Table S1). This finding suggests that the enhancement effect of BHB on intestinal epithelial proliferation could be largely attributed to BHB’s upregulating Arg-1in M2 macrophages.

There are various exogenous ketone supplements, containing BHB salt, BHB ester, medium chain triglycerides or their combinations [23, 49, 50]. Many studies have linked BHB with intestinal homeostasis. A previous research has shown that exogenous BHB supplement exerts anti-aging effect on intestinal stem cells by reducing oxidative stress-induced DNA damage accumulation [51]. The other study has reported that exogenous BHB supplement alters intestinal stem cell numbers and notch activity to regulate intestinal tissue remodeling [18]. Though these researchers have no direct proof that BHB was found in intestinal lumen, exogenous BHB was believed to actually function in the intestinal tract. In a separate study, BHB content was found in the media of mouse intestinal organoid cultures, which suggests that intestinal-derived BHB could be secreted out of cells [52]. More importantly, the recent research has reported that host production of ketone bodies in intestinal lumen could directly induce microbiota shifts [27]. These discoveries provided very important context to our enema experiments. In the present study, we use BHB salt, the most commonly used and effective exogenous ketone supplement, as supplement intervention to treat DSS-induced colitis. To make sure the supplement intervention could accurately and directly impact on intestine of DSS model, BHB salt was given through therapeutic enema. Recent studies indicate BHB monoester could serve as a salt-free precursor to quickly and accurately raise ketone concentration which provides a more safe and healthy application for BHB supplement [29, 49]. Thus, the effect of BHB monoester on DSS-induced colitis merits further studies.

Prior research indicated that ketogenic states, induced by ketogenic diet or fasting, effectively drive BHB generation both in liver and intestine. Increased crypt BHB level and intestinal stem cells (ISCs) numbers and function was observed in mice fed with ketogenic diet. Notably, HMGCS2-derived BHB in ISCs enhances Notch signaling pathway through suppress activity of class I histone deacetylase (Class I HDAC). The Notch transcriptional program in ISCs dynamically modulates ISC self-renewal and intestinal post-injury regeneration in response to diverse physiological states. These results demonstrated convincingly that intestinal BHB serve as a vital signaling molecule to promote intestinal tissue repair processes through maintaining intestinal stemness [18]. Interestingly, another recent study has provided new evidences for the potential role of ketone in intestinal inflammation through the regulation of gut microbiota and intestinal pro-inflammatory Th17 cells. Ketogenic diet exposure significantly altered gut microbial community structure, which was characterized by notably decreased *Bifidobacterium*. Further experiment proved that BHB, generated during ketogenic diet, directly inhibit growth of *bifidobacterial*, which thereby impact immune responses through regulating intestinal Th17 cells populations. These observations together suggested that gut microbial mediated immunomodulation as a mechanism underlying the anti-inflammatory effect of BHB [27]. Increased Th17 immune responses are associated with many autoimmune diseases, including IBD. Th17 cells activation could aggravate intestinal inflammation through expressing IL-17 together with other pro-inflammatory cytokines. Besides, intestinal stemness is essential for tissue remodeling following colitis development. These prominent studies proposed other possibilities that gut microbiota, immune cells, and intestinal stemness modulated by BHB might also contribute to the beneficial effect of BHB on experimental colitis.

## Conclusion

In the present study, we showed that BHB promotes M2 macrophage polarization through the STAT6-dependent signaling pathway, which contributes to the resolution of intestinal inflammation and the repair of damaged intestinal tissues. Based on our finding, a hypothesis for the role of BHB in the pathogenesis of IBD is proposed (Additional file 1: Fig. S7), in which decreased epithelium-derived BHB reduces M2 macrophage polarization, resulting in the decline of tissue repair and anti-inflammatory effect, whereas exogenous BHB supplement reinforces the two effects through enhancing M2 macrophage polarization. Thus, exogenous BHB supplement may offer a useful therapeutic approach for IBD treatment.

## Supplementary Information

**Additional file 1: Table S1.** BHB significantly increases the expression of Arg-1, TGFb2, TGFb3, Wnt5a and Wnt10b in IL-4-stimulated BMDMs. **Table S2.** Demographic characteristics of the study population. **Table S3.** Primer sequences for real-time PCR. **Figure S1.** BHB is decreased in the colons of DSS-exposed mice. **Figure S2.** Exogenous BHB intervention does not affect intestinal epithelial barrier. **Figure S3.** Exogenous BHB intervention affect intestinal microbiota composition. **Figure S4.** The protective effect of BHB against colitis is not depend on gut microbiota. **Figure S5.** BHB alleviates DSS-induced colitis through the STAT6-dependent signaling pathway. **Figure S6.** BHB promotes STAT6 phosphorylation in DSS-induced colitis. **Figure S7.** Model of how beta-hydroxybutyrate (BHB) signaling regulates intestinal macrophage M2 polarization in IBD.

## Data Availability

The data sets supporting the conclusions of this article are listed as follow: https://www.proteinatlas.org; https://platform.opentargets.org. The raw metagenome sequencing data of 16S rDNA reported in this paper have been deposited in the Sequence Read Archive database with the accession numbers: PRJNA812633. The raw metagenome sequencing data of RNA-seq reported in this paper have been deposited in the Genome Sequence Archive database with the accession numbers: CRA006352.

## Abbreviations

ACAT1: Acetyl coenzyme A acetyltransferase 1
Arg-1: Arginase 1
BDH1: β-Hydroxybutyrate dehydrogenase
BHB: Ketone body β-hydroxybutyrate
BMDMs: Bone marrow-derived macrophages
Brdu: 5-Bromodeoxyuridinc
CCK-8: Cell Counting Kit-8
CD: Crohn’s disease
Chil3: Chitinase-3-like protein 1
DSS: Dextran sulfate sodium
ESR: Erythrocyte sedimentation rate
FGF2: Fibroblast growth factor 2
FGF7: Fibroblast growth factor 7
HDAC: Histone deacetylase
HMGCL: HMG-CoA lyase
HMGCS2: 3- Hydroxy-3-methylglutaryl-coenzyme A synthase 2
IBD: Inflammatory bowel disease
IECs: Intestinal epithelial cells
IL: Interleukin
IL-10R: IL-10 receptor
IL-4Ra: IL-4 receptor α
IOD: Integrated optical density
ISCs: Intestinal stem cells
JAK2: Janus kinase2
LPS: Lipopolysaccharide
Nlrp3: NOD-like receptor protein 3
NOS2: Nitric oxide synthase 2
PCNA: Proliferating cell nuclear antigen
PDGFRα: Platelet-derived growth factor receptor α
PDGFRβ: Platelet-derived growth factor receptor β
STAT: Signal Transducer and Activator of Transcription
TGF-β: Transforming growth factor-β
TNF-α: Tumor necrosis factor α
UC: Ulcerative colitis
ZO: Zonula occludens
