# Supplementary material for: Ketone body β-hydroxybutyrate ameliorates colitis by promoting M2 macrophage polarization through the STAT6-dependent signaling pathway
Source: BMC Med. 2022 Apr 15;20:148. doi: 10.1186/s12916-022-02352-x (PMC9011974; doi:10.1186/s12916-022-02352-x)
Supplement: Supplementary file 1 — Additional file 1: Table S1. BHB significantly increases the expression of Arg-1, TGFb2, TGFb3, Wnt5a and Wnt10b in IL-4-stimulated BMDMs. Table S2. Demographic characteristics of the study population. Table S3. Primer sequences for real-time PCR. Figure S1. BHB is decreased in the colons of DSS-exposed mice. Figure S2. Exogenous BHB intervention does not affect intestinal epithelial barrier. Figure S3. Exogenous BHB intervention affect intestinal microbiota composition. Figure S4. The protective effect of BHB against colitis is not depend on gut microbiota. Figure S5. BHB alleviates DSS-induced colitis through the STAT6-dependent signaling pathway. Figure S6. BHB promotes STAT6 phosphorylation in DSS-induced colitis. Figure S7. Model of how beta-hydroxybutyrate (BHB) signaling regulates intestinal macrophage M2 polarization in IBD. [file 12916_2022_2352_MOESM1_ESM.docx]

**Ketone body β-hydroxybutyrate ameliorates colitis by promoting** **M2 macrophage polarization through the STAT6-dependent signaling pathway**

**Chongyang Huang**^1, 2^, **Jun Wang**^1, 2^, **Hongbin Liu**^1, 2^, **Ruo Huang**^1^, **Xinwen Yan**^1^, **Mengyao Song**^1^, **Gao Tan**^1, *^, **Fachao Zhi**^1, 3, *^

^1^Guangdong Provincial Key Laboratory of Gastroenterology, Institute of Gastroenterology of Guangdong Province, Department of Gastroenterology, Nanfang Hospital, Southern Medical University, Guangzhou 510515, China

^2^These authors contributed equally to this study

^3^Lead Contact

^*^Correspondence and requests for materials should be addressed to F.Z. [(zhifc41532@163.com)](mailto:(zhifc41532@163.com)) and G.T. (tgao0316@163.com)

**Table S1 BHB significantly increases the expression of *Arg-1*, *TGFb2*, *TGFb3*,**

***Wnt5a*, and *Wnt10b* in IL-4-stimulated BMDMs**

| Gene  id | Gene  symbol | FPKM  BHB_1 | FPKM  BHB_2 | FPKM  BHB_3 | FPKM  Ctrl_1 | FPKM  Ctrl_2 | FPKM  Ctrl_3 | deseq2_pvalue  BHB vs Ctrl |
| --- | --- | --- | --- | --- | --- | --- | --- | --- |
| 11846 | Arg1 | 163.96 | 140.4 | 82.57 | 42.88 | 9.57 | 33.18 | 0.00048 |
| 11847 | Arg2 | 4.28 | 5.12 | 3.93 | 2.86 | 5.69 | 2.44 | 0.294696 |
| 21802 | Tgfa | 0 | 0 | 0 | 0 | 0 | 0 | NA |
| 21803 | Tgfb1 | 155.48 | 166.12 | 155.37 | 151.8 | 158.42 | 153.22 | 0.255791 |
| 21808 | Tgfb2 | 0.32 | 0.75 | 0.72 | 0.27 | 0.31 | 0.4 | 0.019087 |
| 21809 | Tgfb3 | 0.42 | 0.68 | 0.47 | 0.21 | 0.21 | 0.31 | 0.012344 |
| 22408 | Wnt1 | 0 | 0 | 0 | 0 | 0 | 0 | NA |
| 22413 | Wnt2 | 0 | 0.03 | 0 | 0 | 0 | 0 | 0.808026 |
| 22414 | Wnt2b | 0.2 | 0.33 | 0.1 | 0.2 | 0.27 | 0.26 | 0.761008 |
| 22417 | Wnt4 | 0.07 | 0.03 | 0.2 | 0.08 | 0.06 | 0.18 | 0.890935 |
| 22418 | Wnt5a | 0.25 | 0.27 | 0.26 | 0.13 | 0.03 | 0.15 | 0.009928 |
| 22419 | Wnt5b | 0 | 0.03 | 0 | 0.03 | 0 | 0.03 | 0.768655 |
| 22420 | Wnt6 | 0.21 | 0.37 | 0.18 | 0.24 | 0.15 | 0.18 | 0.508445 |
| 22421 | Wnt7a | 0 | 0 | 0 | 0 | 0 | 0 | NA |
| 216795 | Wnt9a | 0.09 | 0.07 | 0.05 | 0.02 | 0.02 | 0.05 | 0.283428 |
| 22412 | Wnt9b | 0.04 | 0.04 | 0 | 0.09 | 0.11 | 0.12 | 0.050605 |
| 22409 | Wnt10a | 0.03 | 0 | 0 | 0 | 0 | 0 | 0.808026 |
| 22410 | Wnt10b | 0.08 | 0.15 | 0.1 | 0 | 0 | 0.03 | 0.016711 |
| 22411 | Wnt11 | 0.17 | 0.42 | 0.11 | 0.13 | 0.22 | 0.17 | 0.773818 |
| 93735 | Wnt16 | 0.04 | 0 | 0 | 0.01 | 0 | 0.07 | 0.582893 |

Note: BMDMs isolated from mice were stimulated with IL-4 or IL-4 plus BHB. Forty-eight hours later, differentially expressed genes were analyzed by RNA-sequencing. Ctrl represents the IL-4 group; BHB represents the IL-4 plus BHB group.

**Table S2 Demographic characteristics of the study population**


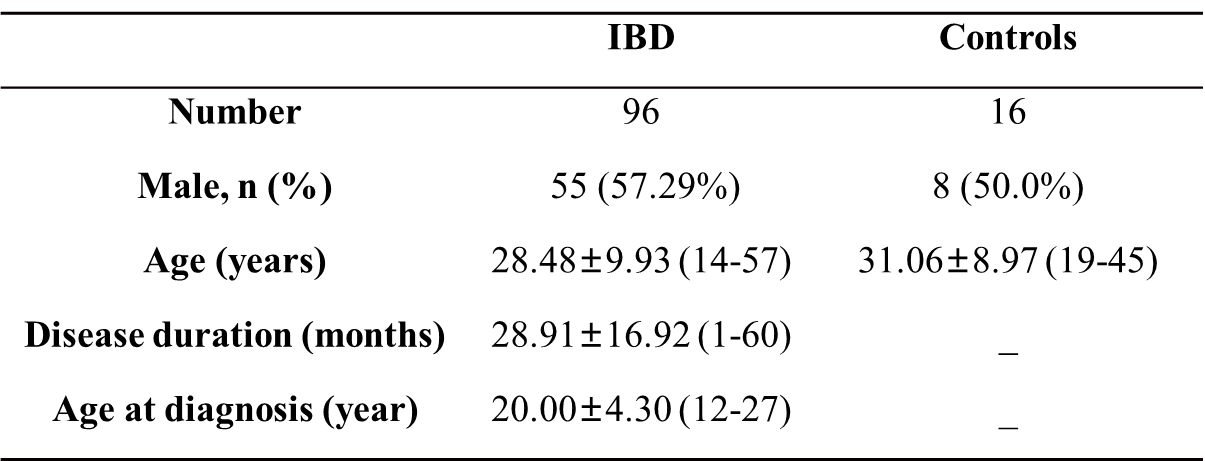


**Table S3 Primer sequences for real-time PCR**

| **Gene** | **Fw_sequence** | **Rv_sequence** |
| --- | --- | --- |
| Arg-1 | AGCTCTGGGAATCTGCATGG | ATGTACACGATGTCTTTGGCAGATA |
| Chil3 | GATGGCCTCAACCTGGACTG | CGTCAATGATTCCTGCTCCTG |
| IL-10 | GCCAGAGCCACATGCTCCTA | GATAAGGCTTGGCAACCCAAGTAA |
| IL-4Ra | TCTGCATCCCGTTGTTTTGC | GCACCTGTGCATCCTGAATG |
| Retnla | CAGCTGATGGTCCCAGTGAA | CAAGCACACCCAGTAGCAGTC |
| IL-12p40 | GCACCTTACACCTACCAGAGT | AAACTTCTGCCTGACGAGCTT |
| TNF-α | ACTCCAGGCGGTGCCTATGT | GTGAGGGTCTGGGCCATAGAA |
| IL-6 | CAACGATGATGCACTTGCAGA | CTCCAGGTAGCTATGGTACTCCAGA |
| NOS2 | TAGGCAGAGATTGGAGGCCTTG | GGGTTGTTGCTGAACTTCCAGTC |
| ACAT1 | CAGGAAGTAAGATGCCTGGAAC | TTCACCCCCTTGGATGACATT |
| HMGCS2  BDH1 | GAAGAGAGCGATGCAGGAAAC  TTCCCCTTCTCCGAAGAGC | GTCCACATATTGGGCTGGAAA  CCCAGAGGGTGCATCTCATAG |
| GAPDH  FGF2  FGF7  TGFb1  TGFbr1  PDGFra  PDGFrb | TGTGTCCGTCGTGGATCTGA  GGAGGGCTGCTGGCTTCTAA  GAACAAAAGTCAAGGAGCAACC  CTCCCGTGGCTTCTAGTGC  TCTGCATTGCACTTATGCTGA  TCCATGCTAGACTCAGAAGTCA  TTCCAGGAGTGATACCAGCTT | TTGCTGTTGAAGTCGCAGGAG  CCAGTTCGTTTCAGTGCCACATAC  GTCATGGGCCTCCTCCTATT  GCCTTAGTTTGGACAGGATCTG  AAAGGGCGATCTAGTGATGGA  TCCCGGTGGACACAATTTTTC  AGGGGGCGTGATGACTAGG |

**
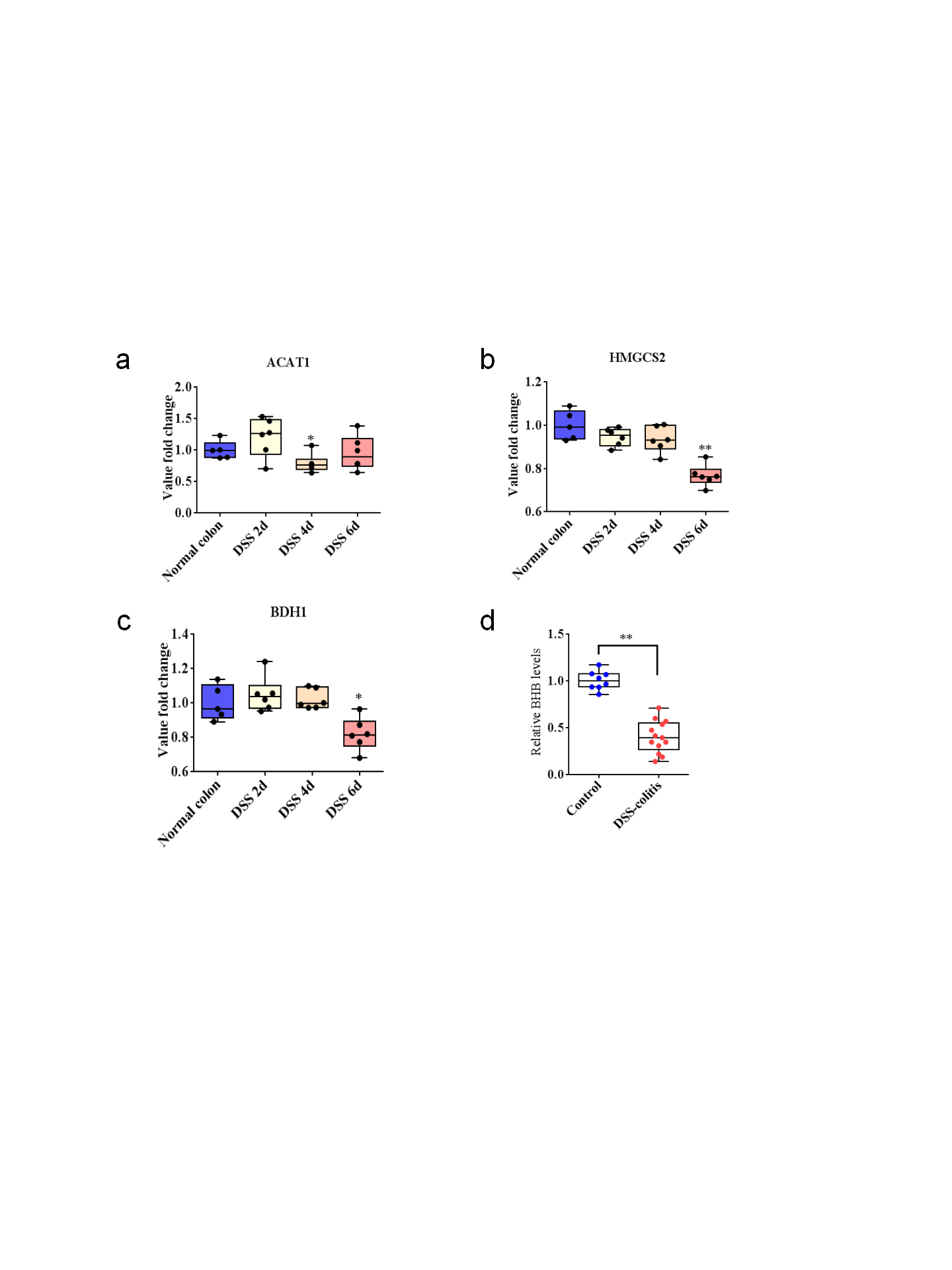
**

**Figure S1 BHB is decreased in the colons of DSS-exposed mice.**

Littermate WT mice were given DSS in drinking water to induce experimental colitis. These mice were killed at the indicated time and their colons were collected for the following analyses.

(a to c) Relative mRNA levels of *HMGCS2*, *ACAT1* and *BDH1* were determined by real-time PCR and normalized to GAPDH.

(d) Enzymatic colorimetric assay of BHB levels in the colons of 6-day DSS-exposed mice. Control represents normal colons untreated with DSS.

Data represent means ± SEM (n =5 to 6 per group); **P* < 0.05, ***P* < 0.01 by unpaired Student’s t test. Data shown are representative of three independent experiments.


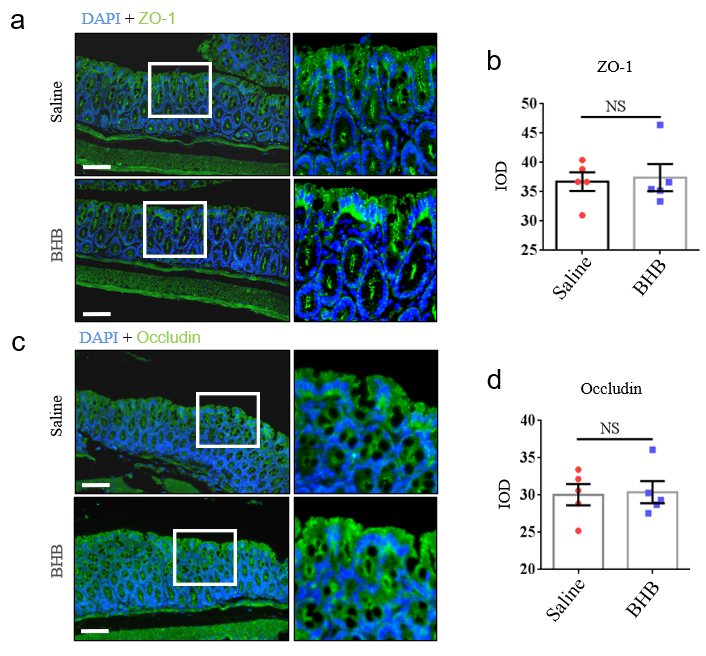


**Figure S2 Exogenous BHB intervention does not affect intestinal epithelial barrier.**

Littermate WT mice were treated with BHB or saline enema. Six days later, these mice killed and their colons were collected for immunostaining analyses.

(a and c) Representative immunofluorescence images of ZO-1 and Occludin immunostaining in colon tissues (scale bars: 100 μm).

(b and d) Quantitative analyses of ZO-1 and Occludin staining by Image-Pro Plus software. IOD: integrated optical density.

Data represent means ± SEM (n = 5 per group); NS, not significant. Unpaired Student’s t test was performed for statistical analysis. Data shown are representative of two independent experiments.


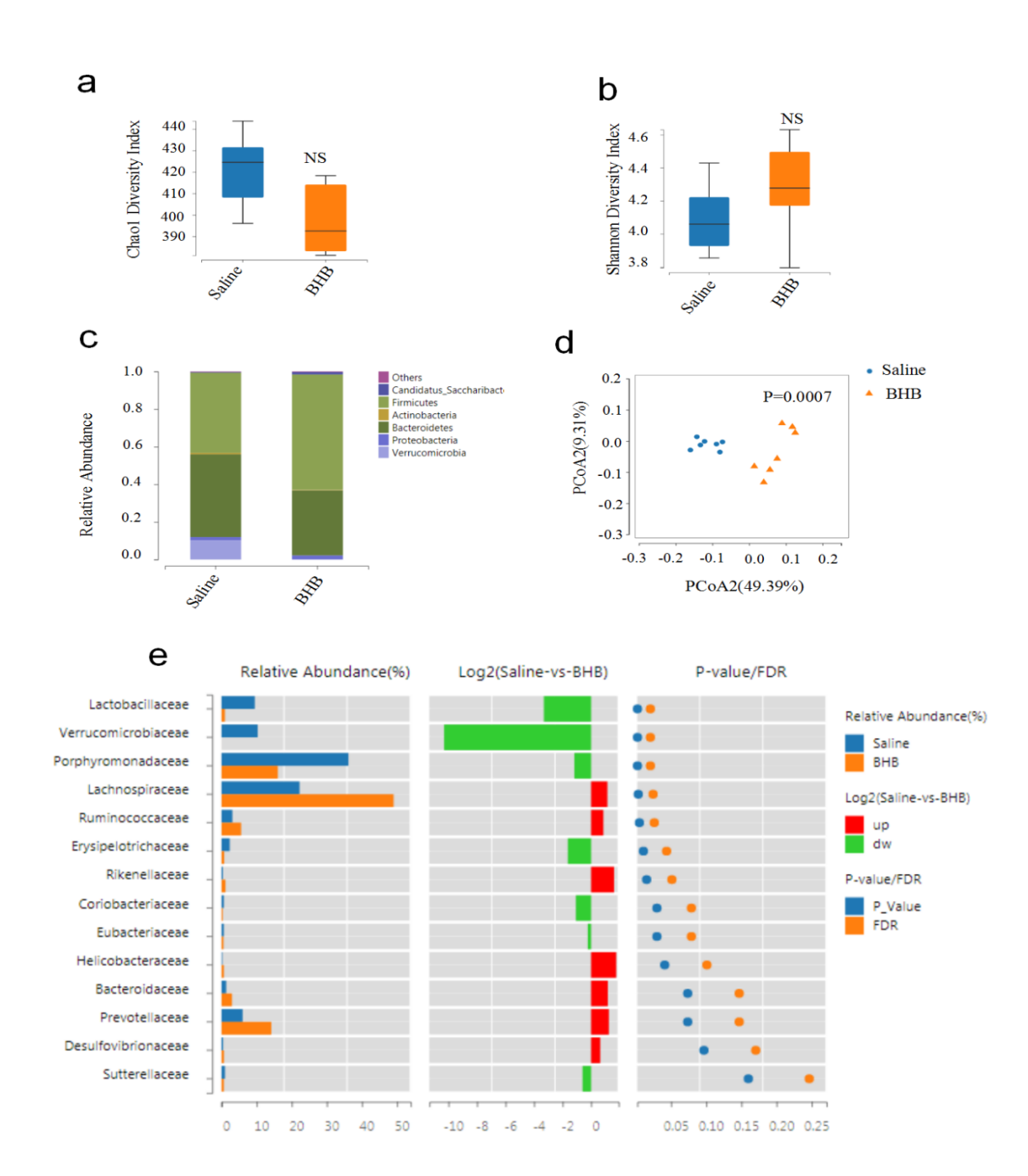


**Figure S3 Exogenous BHB intervention affect intestinal microbiota composition.**

Littermate WT mice were treated with BHB or saline enema. Six days later, feces were collected for 16sRNA analyses.

(a and b) α-diversity Chao1 and Shannon indices of gut microbes.

(c) Relative abundance of gut microbiota in at phylum level.

(d) Scatter plots of weighted PCoA for the microbial composition.

(e) Analysis of species difference in gut microbiota at family level.

Blue box for relative abundance in saline group and orange box for relative abundance in BHB group(left side). Red box for up-regulated species and green box for down-regulated species(middle). Blue dots for P-value and orange dots for False discovery rate (right side).


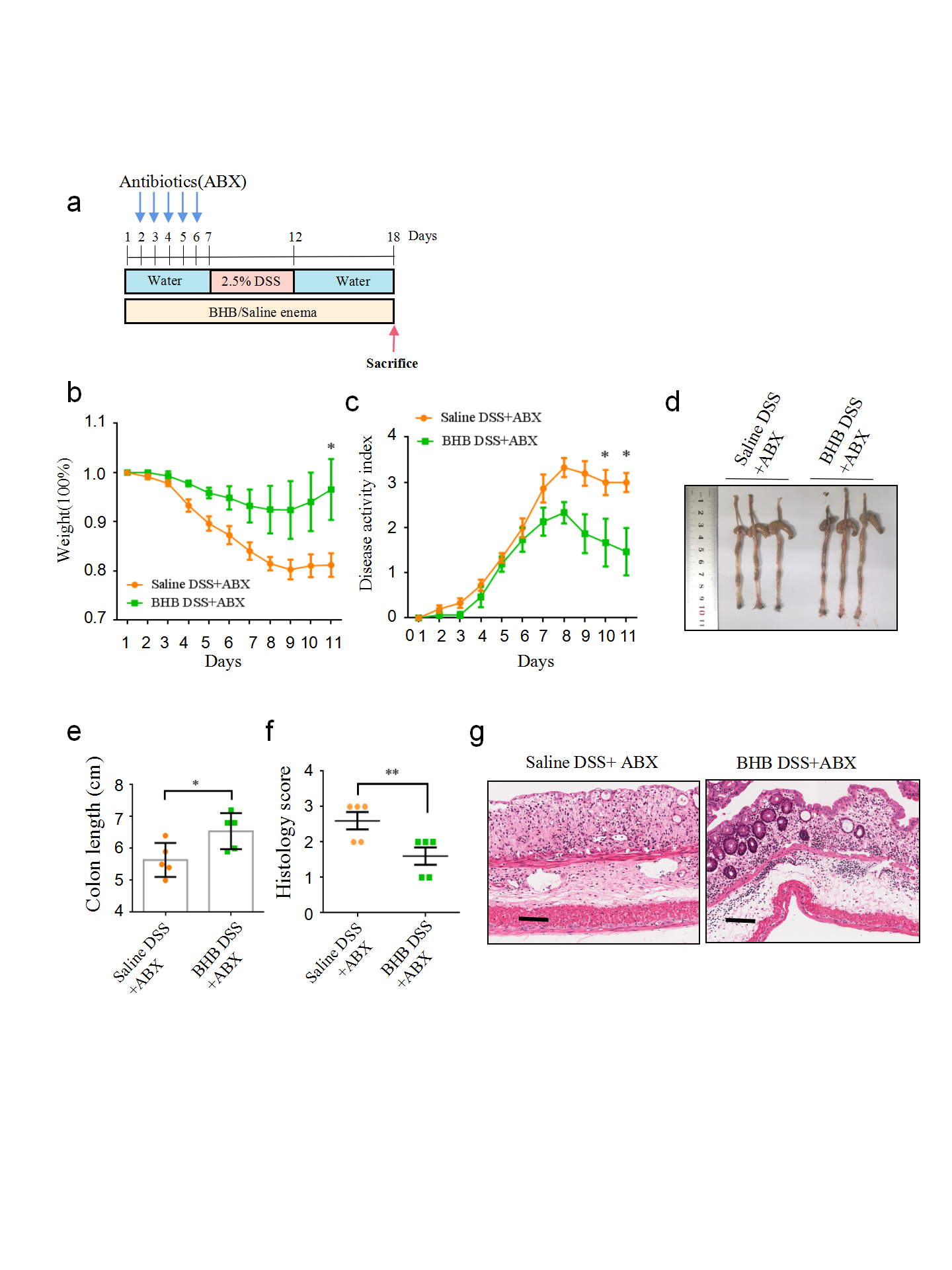


**Figure S4 The protective effect of BHB against colitis is not depend on gut microbiota.**

(a to g) Littermate WT mice received BHB or saline enema were given DSS in drinking water to induce experimental colitis. To ablate gut microbiota, antibiotics (ABX) including 100 mg/kg vancomycin, 200mg/kg neomycin sulfate, 200 mg/kg metronidazole, and 200 mg/kg ampicillin were intragastrically administrated 5 days prior to DSS treatment.

(b and c) Body weight changes and disease activity index were monitored daily after DSS treatment. (d) Representative images of the colons.

(e) Mice were killed on day 18, and colon lengths were measured.

(f and g) Colon sections were examined histologically.

(f) Histology scores for colonic inflammation were measured.

(g) Representative images of the H&E-stained colon sections of different treatment groups (scale bars: 150 μm).

(b, c, e and f) Data represent means ± SEM (n = 5 per group); **P* < 0.05, ***P* < 0.01 by unpaired Student’s t test. Data shown are representative of three independent experiments.


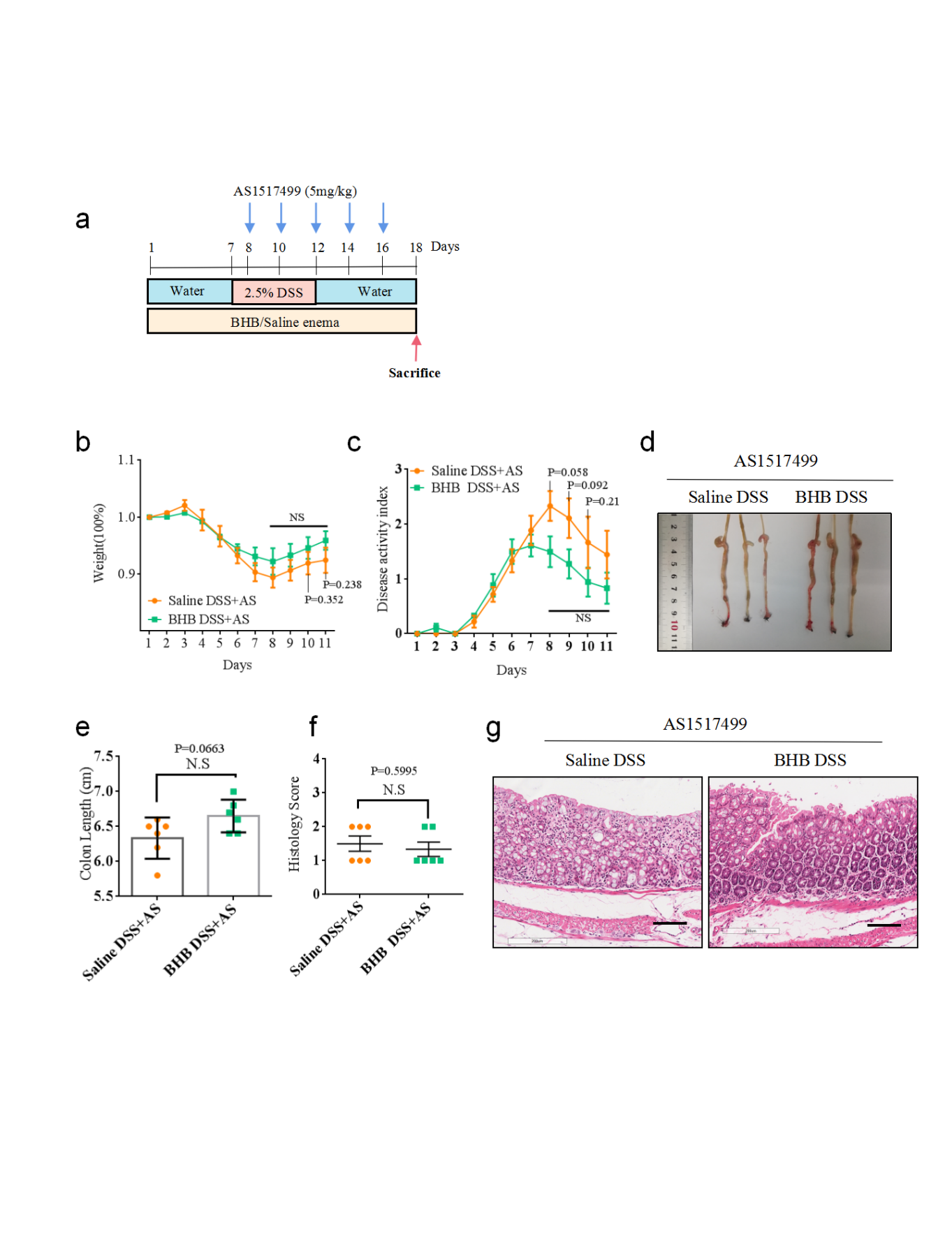


**Figure S5 BHB alleviates DSS-induced colitis through the STAT6-dependent signaling pathway.**

(a to g) Littermate WT mice received BHB or saline enema were given DSS in drinking water to induce experimental colitis. To inhibit STAT6 activation, these mice were intraperitoneally injected with STAT6AS1517499 (5mg/kg), a potent STAT6 phosphorylation inhibitor, on days 1, 3, 5, 7 and 9 after the start of DSS treatment.

(b and c) Body weight changes and disease activity index were monitored daily after DSS treatment.

(d) Representative images of the colons.

(e) Mice were killed on day 18, and colon lengths were measured.

(f and g) Colon sections were examined histologically.

(f) Histology scores for colonic inflammation were measured.

(g) Representative images of the H&E-stained colon sections of different treatment groups (scale bars: 150 μm).

(b, c, e and f) Data represent means ± SEM (n = 6 per group); NS, not significant. Unpaired Student’s t test was performed for statistical analysis. Data shown are representative of two independent experiments.


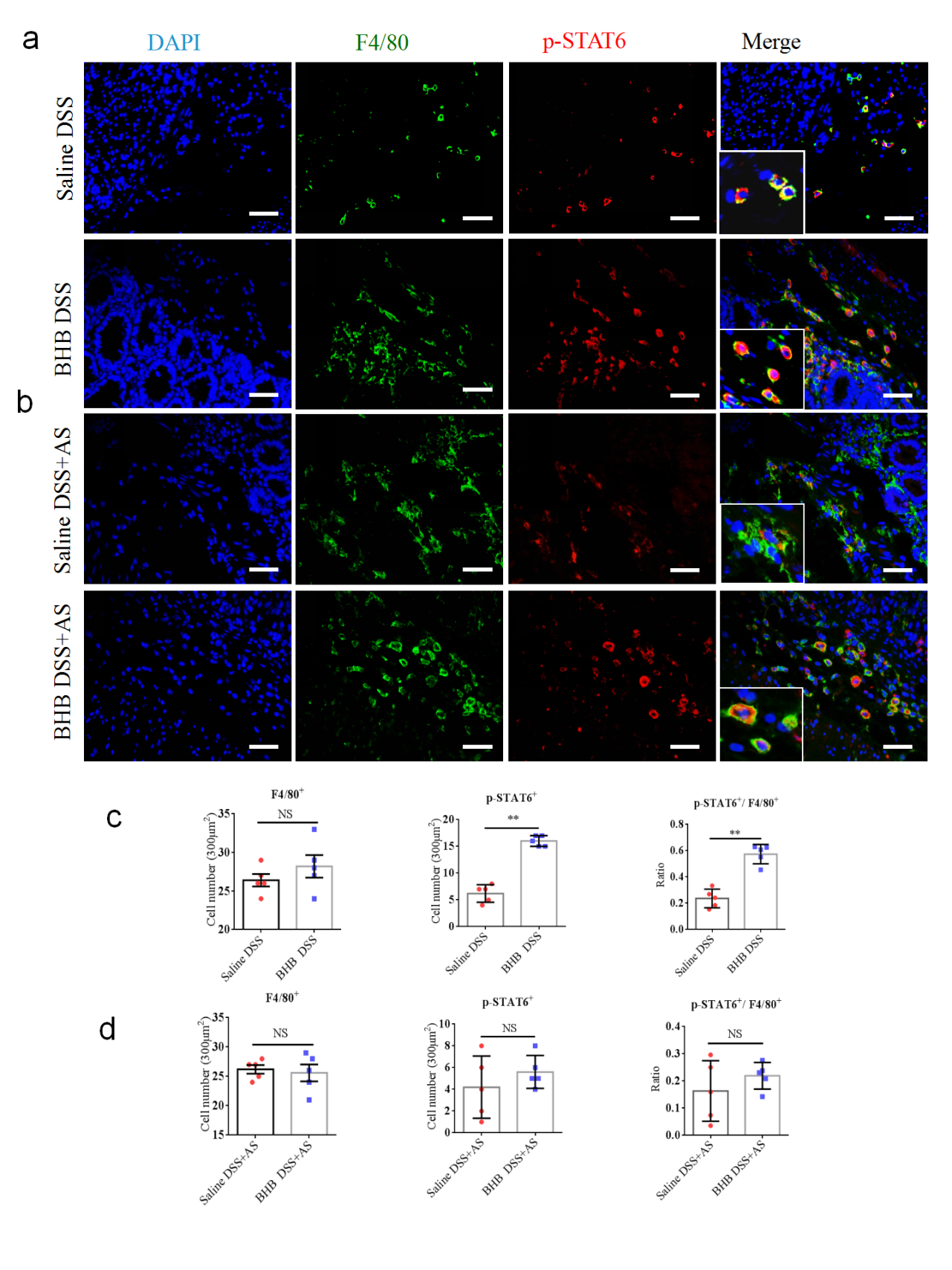


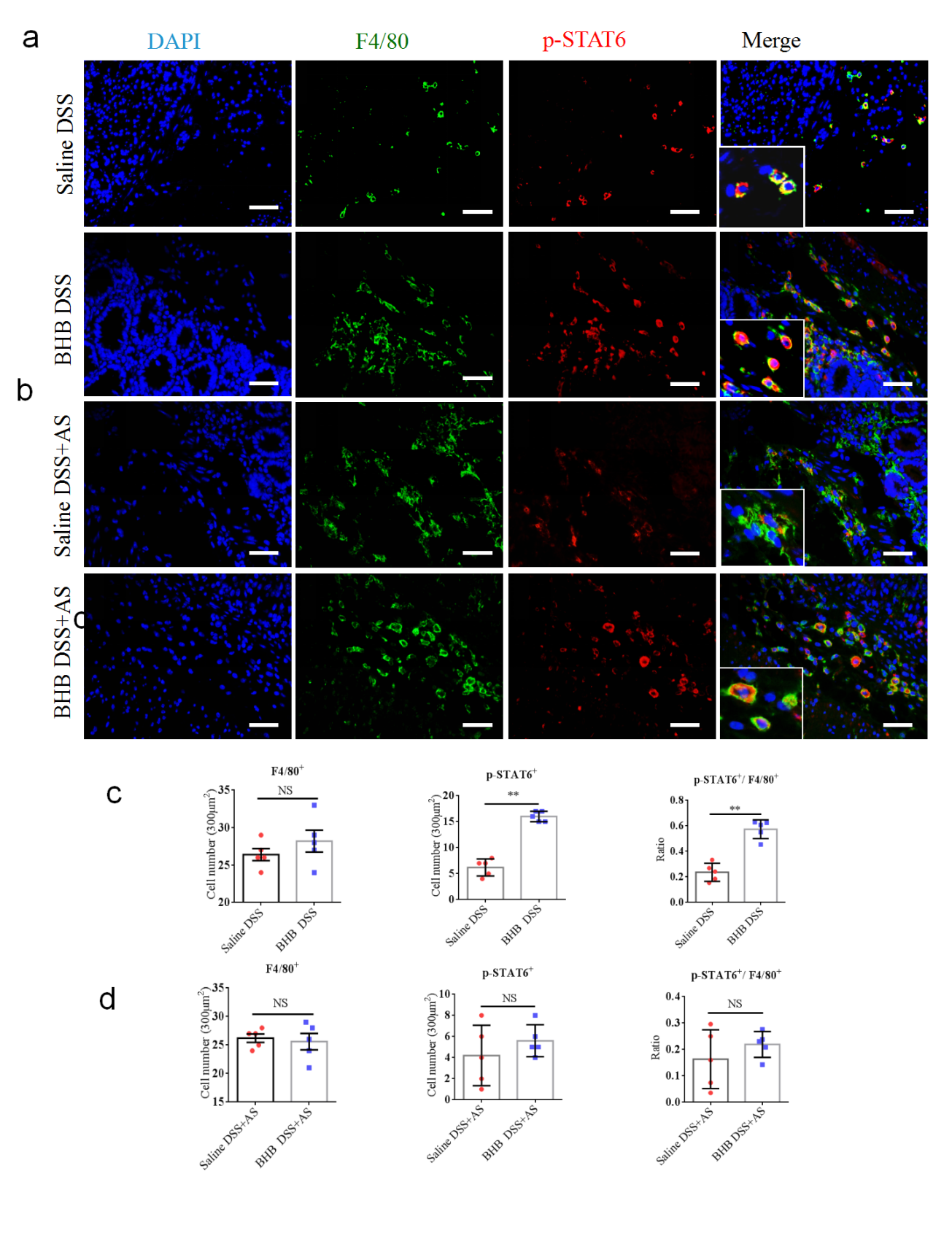


**Figure S6 BHB promotes STAT6 phosphorylation in DSS-induced colitis.**

(a to d) Littermate WT mice received BHB or saline enema were given DSS in drinking water to induce experimental colitis and intraperitoneally injected with STAT6 inhibitor AS1517499 as described in figure S6a. On day 18, these mice were killed and their colon tissues were collected for immunofluorescence analyses.

(a and b) Representative immunofluorescence images of F4/80 and p-STAT6 immunostaining in colon tissues (scale bars: 150 μm). White boxes represent the magnified view.

(c and d) The cell number of F4/80 and p-STAT6 immunostaining per 300 μm^2^. Ratio represents the percentage of p-STAT6^+^ cells in F4/80^+^ cells.

Data represent means ± SEM (n = 5 per group); NS, not significant; ***P* < 0.01 by unpaired Student’s t test. Data shown are representative of three independent experiments.


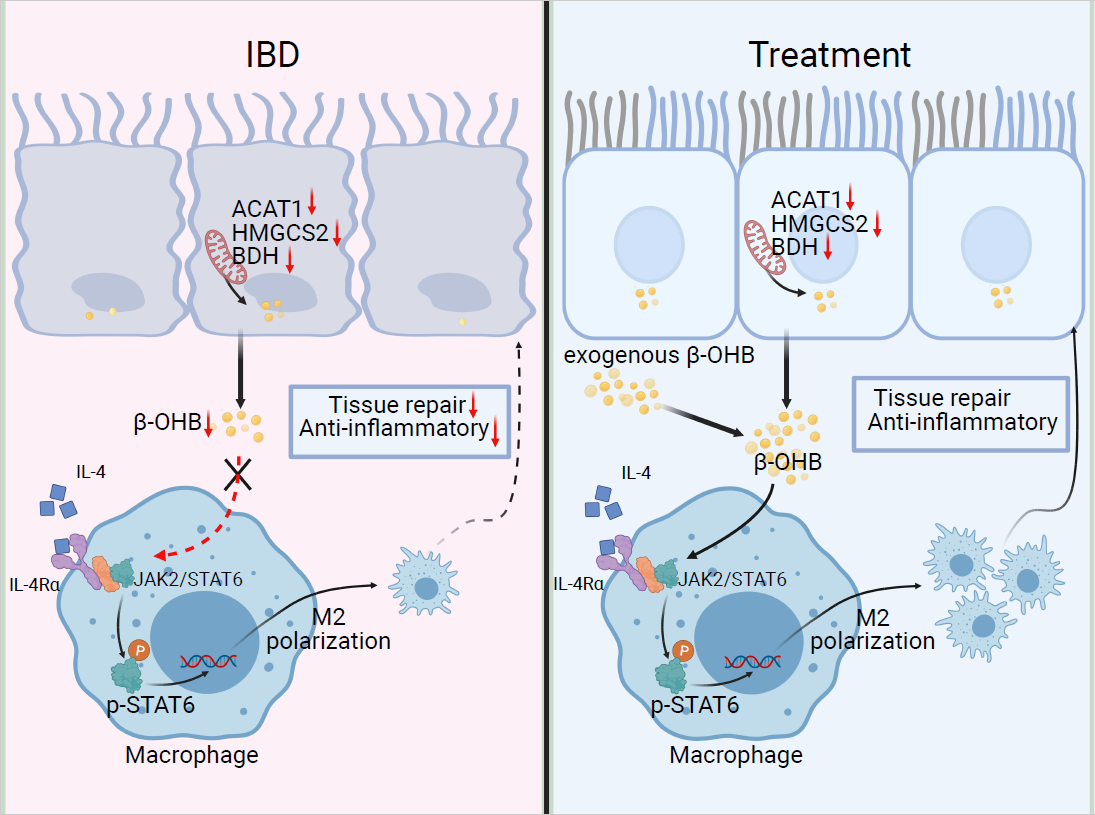


**Figure S7 Model of how β-hydroxybutyrate (βOHB) signaling regulates intestinal macrophage M2 polarization in IBD.**

In this model, decreased epithelium-derived βOHB reduces M2 macrophage polarization induced by IL-4, resulting in the decline of tissue repair and anti-inflammatory effect, whereas exogenous βOHB supplement reinforces the two effects through enhancing M2 macrophage polarization.
